# *Phytophthora sojae* Avirulence Effector Avr3b is a Secreted NADH and ADP-ribose Pyrophosphorylase that Modulates Plant Immunity

**DOI:** 10.1371/journal.ppat.1002353

**Published:** 2011-11-10

**Authors:** Suomeng Dong, Weixiao Yin, Guanghui Kong, Xinyu Yang, Dinah Qutob, Qinghe Chen, Shiv D. Kale, Yangyang Sui, Zhengguang Zhang, Daolong Dou, Xiaobo Zheng, Mark Gijzen, Brett M. Tyler, Yuanchao Wang

**Affiliations:** 1 College of Plant Protection, Nanjing Agricultural University, Nanjing, China; 2 Key Laboratory of Integrated Management of Crop Diseases and Pests (Nanjing Agricultural University), Ministry of Education, Nanjing, China; 3 Agriculture and Agri-Food Canada, London, Ontario, Canada; 4 Virginia Bioinformatics Institute, Blacksburg, Virginia, United States of America; Ohio State University, United States of America

## Abstract

Plants have evolved pathogen-associated molecular pattern (PAMP)-triggered immunity (PTI) and effector-triggered immunity (ETI) to protect themselves from infection by diverse pathogens. Avirulence (Avr) effectors that trigger plant ETI as a result of recognition by plant resistance (*R*) gene products have been identified in many plant pathogenic oomycetes and fungi. However, the virulence functions of oomycete and fungal Avr effectors remain largely unknown. Here, we combined bioinformatics and genetics to identify *Avr3b*, a new *Avr* gene from *Phytophthora sojae*, an oomycete pathogen that causes soybean root rot. *Avr3b* encodes a secreted protein with the RXLR host-targeting motif and C-terminal W and Nudix hydrolase motifs. Some isolates of *P. sojae* evade perception by the soybean *R* gene *Rps*3b through sequence mutation in Avr3b and lowered transcript accumulation. Transient expression of *Avr3b* in *Nicotiana benthamiana* increased susceptibility to *P. capsici* and *P. parasitica*, with significantly reduced accumulation of reactive oxygen species (ROS) around invasion sites. Biochemical assays confirmed that Avr3b is an ADP-ribose/NADH pyrophosphorylase, as predicted from the Nudix motif. Deletion of the Nudix motif of Avr3b abolished enzyme activity. Mutation of key residues in Nudix motif significantly impaired Avr3b virulence function but not the avirulence activity. Some Nudix hydrolases act as negative regulators of plant immunity, and thus Avr3b might be delivered into host cells as a Nudix hydrolase to impair host immunity. Avr3b homologues are present in several sequenced *Phytophthora* genomes, suggesting that *Phytophthora* pathogens might share similar strategies to suppress plant immunity.

## Author Summary

*Phytophthora*, a group of notorious oomycete pathogens, damages a very wide range of crop, vegetable, pasture and horticultural plants, generating great losses to agricultural production annually. Disease outcomes between plants and *Phytophthora* pathogens often depend on whether plants carry resistance (*R*) gene-encoded receptors than recognize the presence of pathogen avirulence (Avr) effectors. Previous studies identified a conserved host-targeting motif, RXLR (arginine, any, leucine, arginine), common to several *Phytophthora* Avr effectors. The genome sequencing of several *Phytophthora* species including *P. infestans* (potato late blight pathogen) and *P. sojae* (soybean root rot disease pathogen), resulted in the identification of a large reservoir of RXLR-carrying effector candidates. In this paper we identified an RXLR-carrying protein from *P. sojae* as Avr effector Avr3b based on genetic mapping, sequence polymorphisms, and transient expression. Avr3b carries a Nudix hydrolase motif at its C-terminus and enhances *Phytophthora* virulence. Biochemical assays revealed that Avr3b is a pyrophosphorylase with ADP-ribose and NADH as its preferred substrates. Furthermore, the enzymatic activity is required for Avr3b to promote virulence but is not required for recognition by Rps3b.

## Introduction

Plant innate immunity, which has been continuously refined by challenges from a diversity of pathogens during evolution, employs at least two defense systems in response to pathogen attacks [1]. One is basal defense through host receptor recognition of conserved pathogen-associated molecular patterns (PAMPs), termed PAMP-triggered immunity (PTI). In most cases, the basal defense response can successfully prevent infections from becoming established. However, successful pathogens deliver secreted proteins (effectors) to suppress basal defense. Therefore, plants have developed a second immunity system that relies on plant resistance (R) protein perception of specific pathogen effectors and is called effector-triggered immunity (ETI). Pathogen effectors recognized by plant R proteins have historically been termed avirulence (Avr) effectors [1], [2]. ETI usually results in faster and stronger plant resistance responses, including programmed cell death (PCD), reactive oxygen species (ROS) accumulation, and induction of plant hormone-mediated signal pathways [1]. Thus, the interaction between host R proteins and pathogen Avr effectors can determine the outcome of an infection.

Many plant *R* genes encode polymorphic proteins characterized by nucleotide binding (NB) and leucine-rich repeat (LRR) domains. These R proteins are located inside the cell, indicating that Avr effectors are usually delivered into plant cells by pathogens [2], [3]. A motif, RXLR (Arg-any-Leu-Arg), was identified in the N-terminus of *Phytophthora* effectors such as *P. sojae* Avr1b and *P. infestans* Avr3a [3], [4], [5], [6] and was experimentally shown to be a host-targeting motif that could translocate effectors into host cells [7], [8], [9]. Recent evidence has shown that *Phytophthora* RXLR effectors could enter host cells without pathogen machinery by binding to the host external lipid phosphatidylinositol-3-phosphate (PI3P), suggesting that the RXLR-PI3P-mediated cell-entering mechanism is used by pathogens to deliver effectors into host cells [10]. Interestingly, nearly all of the Avr effectors identified from oomycetes so far carry the RXLR motif. These Avr effectors include Avr1b, Avr1a, Avr3a/5, Avr3c, and Avr4/6 from *P. sojae*; Avr-blb1, Avr-blb2, Avr2, Avr3a, and Avr4 from *P. infestans*; and ATR1 and ATR13 from the downy mildew pathogen *Hyaloperonospora arabidopsidis* [3], [11], [12], [13], [14], [15], [16], [17], [18], [19], [20]. Although a recently cloned *H. arabidopsidis* avirulence effector, ATR5, does not employ a strict RXLR motif [21], RXLR motifs are still considered to be characteristic signatures of oomycete Avr effectors. Based on the genome sequencing of several oomycete plant pathogens, a large number of RXLR effector candidates were predicted. Altogether, more than 1200 RXLR effector candidates were predicted in the genomes of *P. sojae*, *P. infestans*, *P. ramorum*, and *H. arabidopsidis* [5], [6], [22], [23]. The RXLR motif and the large collection of predicted RXLR effector candidates provide a good resource for Avr effector identification.

Avr effectors are assumed to be important virulence factors that function by manipulating host immunity. However, only a few fungal and oomycete effectors have been functionally characterized [1], [2]. In the fungal pathogen *Cladosporium fulvum*, the Avr effector Avr2 is a cysteine protease inhibitor, and Avr4 acts as a chitin-binding protein that protects *C. fulvum* against host chitinases [24], [25]. The Avr-Pita effector is a zinc-dependent metalloprotease that is required by the fungus *Magnaporthe oryzae* for full virulence on rice [26], [27]. The polyketide synthase activity of *M. oryzae* Ace1 is required for avirulence [27]. In the flax rust *Melampsora lini*, AvrP123 effectors contain a Kazal protease inhibitor signature, but the activity has not been verified [28]. Recently, *Fusarium oxysporum* f. sp. *lycopersici* Avr1 was found to suppress Avr2- and Avr3-triggered ETI in tomato [29]. Avr1b and many other effectors from *P. sojae* could abolish cell death triggered in plants by BAX (a mammalian pro-apoptotic factor), by the PAMP infestin1 (INF1) and by several effectors [30], [31]. Downy mildew *H. arabidopsidis* Avr effectors ATR1 and ATR13 could enhance bacterial virulence on susceptible hosts, while some ATR13 alleles could suppress bacterial PAMP-triggered callose deposition or reduce PAMP-triggered ROS production [32]. *Phytophthora infestans* Avr3a could interact with and stabilize host U-box E3 ligase CMPG1, preventing it from being degraded by the proteasome system and subsequently blocking triggered cell death triggered by INF1 [33]. However, the virulence mechanism of most oomycete and fungal Avr effectors remains to be further explored.

Here, we identify a new oomycete Avr effector, Avr3b, from *P. sojae* that is recognized by soybean plants containing the *R* gene *Rps*3b. Biochemical assays validate the bioinformatic prediction that Avr3b has ADP-ribose/NADH pyrophosphorylase activity. Avr3b contributes to virulence through suppression of plant immunity and that suppression is dependent on enzyme activity. However the enzyme activity of Avr3b is not required for recognition by *Rps*3b-containing plants.

## Results

### RXLR effector candidates for Avr3b

Briefly summarized, previous studies have described the following features of Avr effectors [12], [13], [14]. (1) The transcripts of Avr effectors are detectable in avirulent strains; (2) all of the *Phytophthora* Avr effectors cloned so far possess the RXLR motif; and (3) many Avr proteins are polymorphic. Previous genetic studies also indicated that *Avr3b* segregates as a single dominant gene [34]. Thus, we hypothesized that *Avr3b* was likely encoded by an infection-expressed RXLR effector that is polymorphic among *P. sojae* strains. To identify expressed RXLR effector candidates in avirulent strains, we examined transcription of potential RXLR effector genes in *P. sojae* using digital gene expression (DGE), Affymetrix arrays, and expressed sequence tags (ESTs). The DGE method resulted in the identification of 81 expressed RXLR effector genes [35]. A BLAST search in the *P. sojae* EST database identified 16 expressed RXLR effector genes. After the addition of 59 previously identified expressed RXLR effector genes from microarray analysis [12], [31], a total of 119 RXLR effectors were detected as expressed in the *P. sojae* Avr3b avirulent strain P6497 (Table S1).

Genomic and mitochondrial DNA RFLP results delineated *P. sojae* strains into four major genetic lineages [36]. *Phytophthora sojae* isolates P6497, P7064, P7074, and P7076 are representative isolates from each lineage. In addition to the published genome sequence of P6497, the re-sequencing of three *P. sojae* lineages (P7064, P7074, and P7076) has recently been completed [31]. Investigation of the 131 expressed effector sequences among these lineages revealed that six effectors (Avh20, Avh113, Avh238, Avh258, Avh288, and Avh307) showed a sequence polymorphism pattern consistent with the Avr3b phenotype; namely, identical in P6497 and P7074 (Avr3b avirulent strains) but variant in P7074 and P7076 (virulent strains) (Table 1). Furthermore, Avh6 showed a presence/absence polymorphism between avirulent and virulent strains (Table 1). Thus, these seven effectors were selected as Avr3b candidates for further investigation.

**Table 1:** Seven expressed RXLR effectors showed Avr3b polymorphism.

Name	DGE^a^	Affy^a^	EST^a^	SP HMM value^b^	SP Length^b^	RXLR^c^	dEER^c^	P7064^d^	P7074^d^	P7076^d^
Avh6		+		0.998	25	RFLR	DNEER	Polymorphic	None	None
Avh20		+		1.000	21	RLLR	DNEEER	Identical	Polymorphic	Polymorphic
Avh113	+	+		1.000	20	RFLR	EER	Identical	Polymorphic	Polymorphic
Avh238	+	+	+	0.999	19	RFLR	DGKTEER	Identical	Polymorphic	Polymorphic
Avh258	+			1.000	24	RHLK	DLTAESEER	Identical	Polymorphic	Polymorphic
Avh288	+	+	+	0.999	20	RRLK	DLTAHEEER	Identical	Polymorphic	Polymorphic
Avh307	+			1.000	17	RSLR	EER	Identical	Polymorphic	Polymorphic

**a**: Identification of expressed RXLR effectors by examination of three kinds of gene expression data: DGE, digital gene expression data generated by Solexa sequencing [35]; Affy, soybean Affymetrix array data [12], [31]; and ESTs [61], the expression sequence tags were obtained by BLAST in VBI *P. sojae* unigenes (VBI microbial database). “+” indicates that transcripts of the gene were detectable using the method. All these data were derived from *P. sojae* strain P6497 (*Rps*3b avirulent). **b**: SP HMM (signal peptide prediction HMM probability) and SP length (signal peptide) were predicted using SignalP v3.0 (http://www.cbs.dtu.dk/services/SignalP/). **c**: RXLR and dEER motifs were previously bioinformatically identified [42]. **d**: The sequences of RXLR effectors were obtained from three *P. sojae* lineages (P7064, P7074, and P7076) using 454 genome sequencing assembly (VBI microbial database)[31]; P7064 is an Avr3b avirulent lineage, and P7074 and P7076 are Avr3b virulent lineages. The translated amino acid sequences were compared with RXLR effectors in P6497. Polymorphism results defined as “identical” means that the amino acid sequences were perfectly identical; “none” means that the gene was missing in the genome assembly; and “polymorphic” represents mutations including SNPs, insertion, or deletion.

### Genetic co-segregation of the Avh307 genotype with the Avr3b phenotype

**Figure 1:**
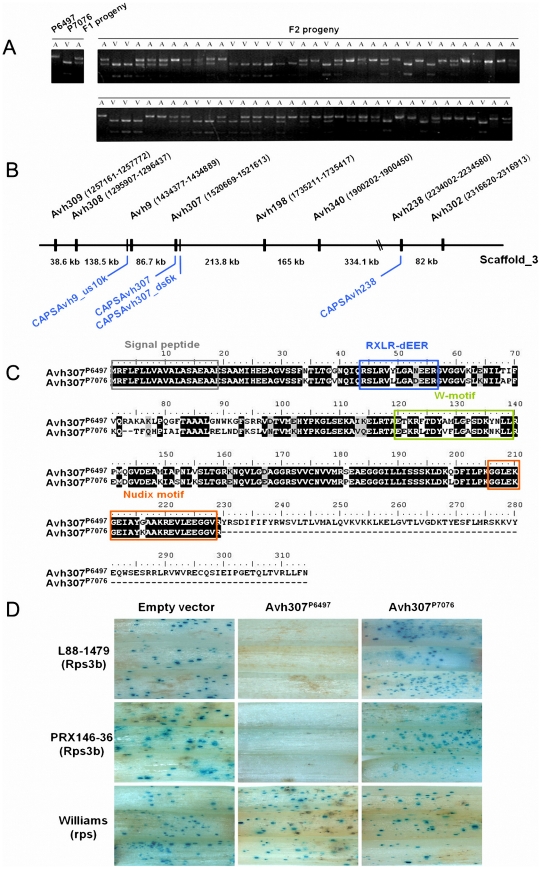
*Avh*307 corresponds to *Avr3b* based on genetic mapping, sequence polymorphisms, and bioassays. (**A**) Co-segregation of *Avh*307 with the Avr3b virulence phenotype in F_2_ progeny from a cross between *P. sojae* isolates P6497 and P7076. A virulence assay was performed with cultures scored as virulent (V) or avirulent (A) on Rps3b soybean plants. Cleaved amplified polymorphic (CAP) markers, co-dominant and specific for Avh307, were scored using genomic DNA from the avirulent (A) and virulent (V) parents and F_1_ and F_2_ progeny. Shown is a photograph of an ethidium bromide-stained agarose gel of the CAP marker results for Avh307. (**B**) Physical map of the *Avh*307 region. Predicted RXLR genes and CAP DNA markers for genetic mapping are shown. Genes (black color) and CAP markers (blue color) are shown on *P. sojae* P6497 genome sequence scaffold_3 (assembly version 5.0), not to scale. (**C**) Predicted amino acid sequences of Avh307^P6497^ (from P6497 and other *Rps*3b-avirulent strains) and Avh307^P7076^ (from P7076 and other *Rps*3b-virulent strains). Predicted signal peptide, RXLR-dEER motif, W-motif, and a Nudix hydrolase motif are shown in grey, blue, green and orange frames. (**D**) Transient expression assays of Avh307 alleles in *Rps*3b plant tissue. Soybean hypocotyls of cultivars Williams (*rps*), L88-1479 (*Rps*3b), and PRX146-36 (*Rps*3b) were transformed by co-bombardment with a plasmid mixture consisting of a glucuronidase reporter gene (GUS) expression vector and a test construct. Expression of the GUS reporter and Avh307 alleles were controlled by the 35S promoter. The test constructs used in combination with the GUS reporter are shown on the top of the panel.

To determine whether candidate effectors might match *Avr3b*, we conducted genetic mapping to identify effectors that co-segregated with the Avr3b avirulence phenotype. First, we crossed P6497 and P7076, and identified 3 F_1_ individuals. All the 3 F_1_ individuals showed avirulence on *Rps*3b soybeans. Then, one F_1_ individual was selected for self-crossing, resulting in a total of 71 F_2_ individuals (*P. sojae* is diploid and homothallic, and wild-type strains show low levels of heterozygosity, hence segregation for most loci occurs in the F_2_ generation). Avirulence assays of these progeny on soybean plants carrying resistance gene *Rps*3b distinguished 51 avirulent and 20 virulent F_2_ individuals. The observed F_2_ population segregation ratio for *Avr3b* fit a 3∶1 ratio (χ^2^  = 0.38, p >0.05), suggesting that the *Avr3b* avirulence phenotype is conferred by a single dominant allele, consistent with a previous report [34]. Cleaved amplified polymorphic (CAP) DNA markers were designed based on the sequence polymorphisms of the candidate effector genes (Table S2). The *Avh*307 genotype was found to perfectly match (0% recombination) the Avr3b avirulence phenotype among the 71 F_2_ progeny (Figure 1A). In contrast, *Avh*238 (8.3% recombination), *Avh*288 (29%), *Avh*20 (33%), *Avh*6 (40%), *Avh*113 (35%), and *Avh*258 (31%) did not match.

*Avh*238 and *Avh*307 are genetically linked and are located on the same genome sequence scaffold, 713 kb apart (*Avh*238, scaffold_3: 2234002–2234580; *Avh*307, scaffold_3: 1520669–1521613) (Figure 1B). Examination of 1.06 Mb of DNA sequence surrounding Avh307 revealed another six RXLR effector genes (*Avh*309, *Avh*308, *Avh*9, *Avh*198, *Avh*340, and *Avh*302) in this region (Figure 1B; Table S3). Analysis by RT-PCR of all of these effector candidates revealed that only *Avh*309 and *Avh*340 were expressed in P6497 during infection (Table S3). However, neither *Avh*309 nor *Avh*340 displayed sequence polymorphisms. The predicted effector gene *Avh*308 displayed polymorphisms but was not expressed during infection. To generate a more detailed genetic map of the *Avr3b* locus, we identified another two markers (CAPSAvh307_ds6k and CAPSAvh9_us10k) in the vicinity of *Avh*307. The marker CAPSAvh307_ds6k (scaffold_3:1515512–1516432) is located 6 kb downstream of *Avh*307; this marker also co-segregated with the Avr3b avirulence phenotype without recombination in our 71 F_2_ progeny. The marker CAPSAvh9_us10 k (scaffold_3: 1424151–1424781) is located 96 kb upstream of *Avh*307; this marker showed 4.4% recombination with the Avr3b avirulence phenotype (Figure 1B). Thus, of the eight RXLR genes examined in this region, *Avh*307 emerged as the best candidate for *Avr3b* (Table S3).

Oomycete Avr effectors often show significant sequence polymorphisms among field populations [12], [13], [14]. We examined *Avh*307 polymorphisms among 20 *P. sojae* field isolates from China. As shown in Figure 1C, *Avh*307 from each of the 16 avirulent strains encoded a 315 amino acid protein identical in sequence to *Avh*307^P6497^. However, the four virulent isolates encoded a protein identical to *Avh*307^P7076^ which is truncated to 230 amino acids by a premature stop codon (Figure 1C). Outside of the truncated region, an additional 46 amino acid substitutions and two deletions were present in *Avh*307^P7076^ compared to *Avh*307^P6497^.

In addition to a secretory signal peptide and RXLR and dEER motifs, the sequence of the *Avh*307^P6497^ allele contains a W motif [30], and a nuclear diphosphate hydrolase (Nudix) motif, G5XE7XREUXEEXGU (Conserved Domain Database ID: cd04666, E-value  = 1.52^e-07^) (Figure 1C). The *Avh*307^P7076^ allele retained all of these motifs but with polymorphic sites in the dEER-, Nudix-, and W-motifs, as illustrated in Figure 1C.

### Expression of Avh307 from avirulent strains triggers *Rps*3b-mediated cell death

The proteins encoded by *P. sojae Avr* genes trigger cell death when they are expressed in or enter into soybean cells containing their matching soybean Rps proteins. To test whether *Avh*307 alleles could trigger cell death in the presence of *Rps*3b, we conducted a soybean transient expression experiment by co-bombardment of soybean hypocotyls to measure cell death [37]. Plasmid constructs carrying *Avh*307 alleles encoding mature proteins without native signal peptides were delivered by particle bombardment into soybean hypocotyls, along with a beta-glucuronidase (GUS) reporter gene to measure cell survival. The *Avh*307^P6497^ allele triggered cell death in the presence of *Rps*3b, but not in the absence of *Rps*3b, as evidenced by a strong reduction in GUS staining. This experiment was performed on two different soybean lines carrying the *Rps*3b gene and on control plants without *Rps*3b (Figure 1D). In contrast, expression of *Avh*307^P7076^ did not reduce GUS staining in the presence of the *Rps*3b gene, nor in its absence (Figure 1D). To further confirm our results, we performed a double-barreled bombardment which could compare cell death in a more direct fashion [30]. These data confirmed that expression of the *Avh*307^P6497^ allele strongly triggered cell death on *Rps*3b soybeans, but no reaction on near-isogenic soybean lines that do not contain *Rps3b* (Figure S1). *Avh*307^P7076^, the virulence allele, did not induce significant cell death on *Rps3b* plants. Thus, both kinds of co-bombardment assays provided functional evidence that *Avh*307 is *Avr3b*, and that *Avh*307^P6497^ and *Avh*307^P7076^ are avirulence and virulence alleles, respectively, of *Avr3b*.

### The *Avr3b* gene is expressed during infection and shows transcriptional polymorphisms between virulent and avirulent strains

**Figure 2:**
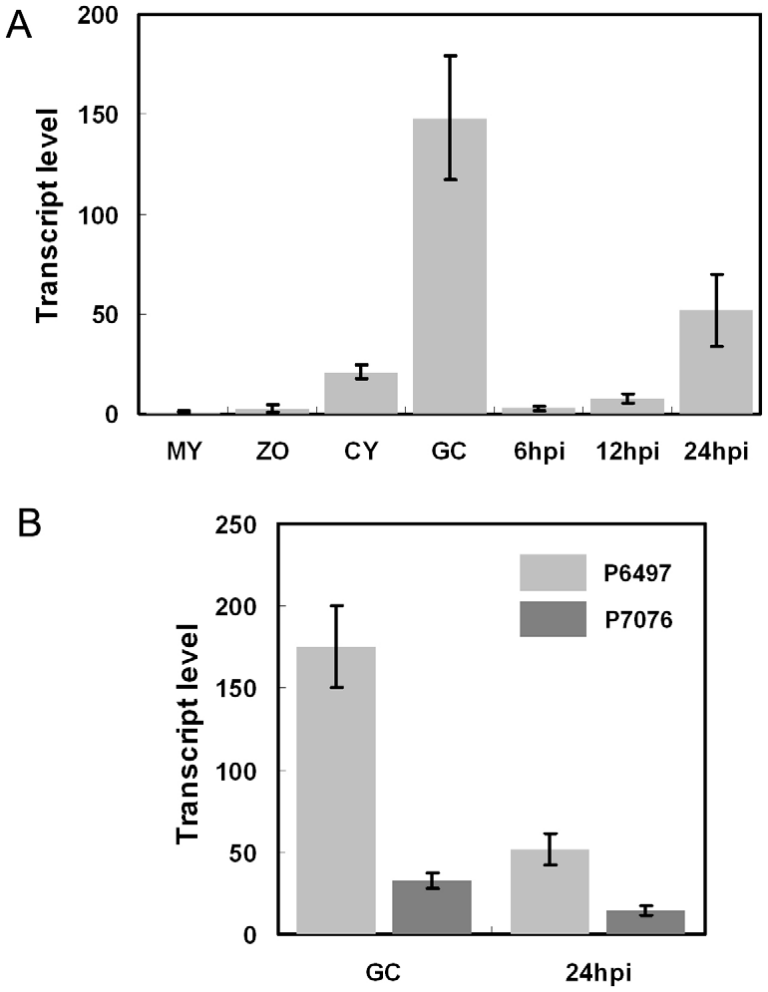
Avr3b is induced during infection and shows transcriptional polymorphisms. (**A**) Avr3b transcript levels in different developmental and infection stages measured by real-time RT-PCR. Total RNA was extracted from mycelia (MY), zoospores (ZO), cysts (CY), germinating cysts (GC), and infected soybean leaves at 6 hours post-infection (hpi), 12 hpi, and 24 hpi. All inoculations for RNA isolation were performed on susceptible soybean cultivar Williams. Real-time RT-PCR analysis employed primers specific for Avr3b and the actin gene. Transcrip level represent the *P. sojae* Avr3b mRNA levels compared with actin mRNA levels. Bars represent standard errors from 2 independent replicates each. (**B**) Avr3b transcriptional polymorphism between virulent (P7076) and avirulent (P6497) strains. Real-time RT-PCR analysis was conducted to compare Avr3b transcript levels at two infection-related stages, germinating cysts (GC) and infected leaves (24 hpi). Primers used in this assay amplified identical regions from Avr3b^P6497^ and Avr3b^P7076^. Bars represent standard errors from 2 independent replicates each.

To explore how *Avr3b* may be involved in *P. sojae*-soybean interactions, we examined the *Avr3b* transcriptional profile at different stages of infection and among different isolates. To define the transcriptional pattern of *Avr3*b, we isolated total RNA from mycelia, zoospores, cysts, germinating cysts, and from infected susceptible soybean leaves at 6 hours post-infection (hpi), 12 hpi, and 24 hpi. Real-time RT-PCR data indicated that *Avr3b* transcript levels are significantly elevated during infection (Figure 2A). Compared with *in vitro* grown mycelia, the *Avr3b* transcript level was elevated 20-fold, 150-fold, and 50-fold in the cyst, germinating cyst, and 24-hpi stages, respectively. These data indicate that Avr3b is strongly induced in pre-infection structures and during infection.

*P. sojae* Avr effector genes *Avr1b, Avr1a,* and *Avr3a* all showed transcriptional polymorphisms among *P. sojae* strains [12], [13], [14]. To test for transcriptional polymorphisms of *Avr3b*, we compared *Avr3b* transcript levels in the germinating cyst and 24-hpi stages between *P. sojae* isolates P6497 (avirulent) and P7076 (virulent) using real-time RT-PCR. In P6497 compared to P7076, *Avr3b* transcript levels were five times higher at the germination cyst stage and three times higher at the 24-hpi stage (Figure 2B), indicating that *Avr3b* exhibits significant transcriptional polymorphism. However, despite the relatively lower expression level, transcripts of the *Avr3b* virulence allele remain detectable, indicating that it might be required during infection.

### Avr3b enhances *Phytophthora* virulence on *N. benthamiana* and decreases ROS accumulation

**Figure 3:**
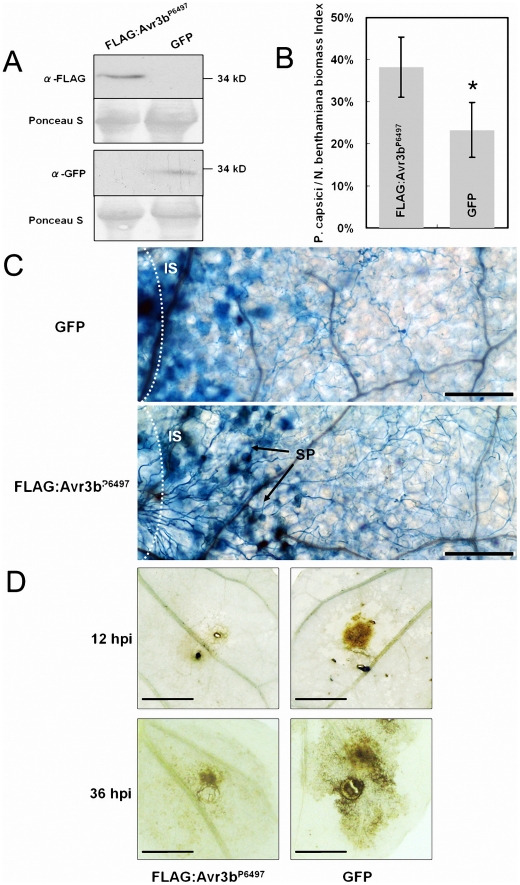
Expression of Avr3b^P6497^ in *N. benthamiana* increased *Phytophthora* susceptibility and suppressed ROS accumulation. (**A**) Western blot assay of Avr3b^P6497^ and GFP protein levels in *N. benthamiana* leaves following *Agrobacterium*-mediated transient expression. Anti-FLAG monoclonal antibody was used to detect levels of FLAG-tagged Avr3b^P6497^ protein. Anti-GFP monoclonal antibody was used to detect GFP protein levels. (**B**) Real-time PCR measurement of pathogen and plant DNA ratios was used to determine *P. capsici* biomass in infected plant tissues following *Agrobacterium*-mediated transient expression of FLAG-Avr3b or GFP. *Nicotiana benthamiana* leaves infiltrated with *Agrobacterium* carrying different constructs were grown in a greenhouse for 48 hours then inoculated with agar plugs containing *P. capsici* mycelium. DNA from *P. capsici* infected regions was isolated at 36 hpi. Real-time PCR employed primers specific for the *N. benthamiana* and *P. capsici* actin genes. Statistical analysis was performed by the Wilcoxon rank sum test (asterisk indicates p<0.01). Bars represent standard errors from 6 replicates (two technical replicates each from three biological replicates). (**C**) Photomicrographs from *P. capsici* infected regions. Leaf regions transiently expressing Avr3b^P6497^ or GFP, and inoculated with *P. capsici* for 16 hours, were stained with trypan blue. Bar  = 2.5 µm. IS: inoculation sites, SP: sporangia. (**D**) ROS generation during *P. capsici* infection following Avr3b^P6497^ or GFP transient expression in *N. benthamiana* leaves at 12 hpi (upper panel) and 36 hpi (lower panel). Leaves were stained with DAB. Bar  = 5 mm. Typical results are shown from 4 replicates.

Several oomycete avirulence effectors can suppress aspects of plant immunity [8], [32], [38]. To determine whether *Avr3b* interferes with plant immunity, we transiently expressed recombinant Avr3b^P6497^ (without a signal peptide) fused with a FLAG tag in *N. benthamiana* using agroinflitration with a potato virus X (PVX) vector. Total protein samples were isolated from agroinfiltrated plant leaves and subjected to western blot analysis, employing an anti-FLAG monoclonal antibody as a probe. The results show expression of a protein of the expected size (34-kDa) in the tissue infiltrated with the Agrobacterium cells carrying the FLAG:Avr3b^P6497^ construct (Figure 3A), consistent with expression of FLAG:Avr3b^P6497^ in *N. benthamiana*.

Two *Phytophthora* pathogens of *N. benthamiana*, *P. capsici* and *P. parasitica,* were used to the challenge the infiltrated tissue. After 48 hours following *Agrobacterium* infiltration, we inoculated the infiltrated regions with an agar plug (5×5 mm) containing freshly grown *P. capsici* and *P. parasitica* mycelia, then evaluated disease development. On leaves infiltrated with strains carrying a control gene (*GFP*), the diameter of the disease lesions was approximately 0.7 to 0.8 cm at 36 hpi; however, on leaves infiltrated with strains carrying *Avr3b* ^P6497^ the lesion diameter expanded to 1.3 to 1.4 cm, as shown in Figure S2. To more precisely measure *P. capsici* infection, we measured the ratio of *P. capsici* DNA to *N. benthamiana* DNA using real-time PCR to determine the *Phytophthora* biomass in the infected plant tissues (Figure 3B). The data show that the *P. capsici*/*N. benthamiana* biomass ratio was significantly higher in Avr3b^P6497^-infiltrated leaves (38%) than in GFP leaves (24%) (P value < 0.01). Both the lesion size data and the biomass data suggest that Avr3b expression *in planta* enhanced the susceptibility of *N. benthamiana* to *Phytophthora*.

To explore possible mechanisms behind the increased *Phytophthora* susceptibility, trypan blue and diaminobenzidine (DAB) staining were performed to examine infected plant tissues for the distribution of hyphae and for ROS production, respectively. At 16 hpi, a higher density of infected mycelium was observed in *Avr3b* ^P6497^-transformed tissues (Figure 3C), consistent with the real-time PCR data showing greater *P. capsici* biomass in *Avr3b* ^P6497^-expressing tissues. Furthermore sporangia had formed within the infected regions of *Avr3b* ^P6497^-expressing leaves, whereas none were found within the infected regions of *GFP*-expressing leaves (Figure 3C). In contrast, there was more extensive trypan blue staining of plant cells in infected tissues expressing the *GFP* control than in those expressing *Avr3b* ^P6497^, suggesting that more defense-related cell death may have occurred in those tissues. Substantially less DAB staining of 16-hpi plant tissues was observed in infected regions of *Avr3b* ^P6497^-expressing leaves compared to the *GFP* control, suggesting that ROS accumulation in response to infection was significantly reduced in the Avr3b^P6497^-expressing tissue (Figure 3D). Overall, transient expression of *Avr3b* ^P6497^ in *N. benthamiana* leaf tissue resulted in a greater degree of *Phytophthora* infection with less ROS accumulation and less cell death, compared to the *GFP* control.

### Avr3b-like RXLR-Nudix proteins are conserved in *Phytophthora* species, but not in other oomycetes

To examine the distribution of *Avr3b* homologs in other oomycete species, we performed a BLAST similarity search in several oomycete genome databases. A search of the *P. sojae* genome identified another 12 predicted genes that had the Nudix motif. A total of 15 Nudix hydrolases were found in the *P. infestans* genome; four of these hydrolases (PITG05846, PITG06308, PITG15679, and PITG15732) had a signal peptide and RXLR motif. The *P. ramorum* genome encoded a total of 14 Nudix hydrolases; three (PrAvh165, PrAvh268, and PrAvh281) were RXLR effectors. Only one RXLR Nudix hydrolase (ID: 102433) was defined in the JGI *P. capsici* genome. We also searched other plant and animal oomycete databases, including *H. arabidopsidis*, *Pythium ultimum*, and *Saprolegnia parasitica* without finding any predicted RXLR Nudix hydrolase effectors, suggesting that this gene family might be present only in the *Phytophthora* genus. Thus, an Avr3b family composed of nine members from four *Phytophthora* species was identified (Table S4).

**Figure 4:**
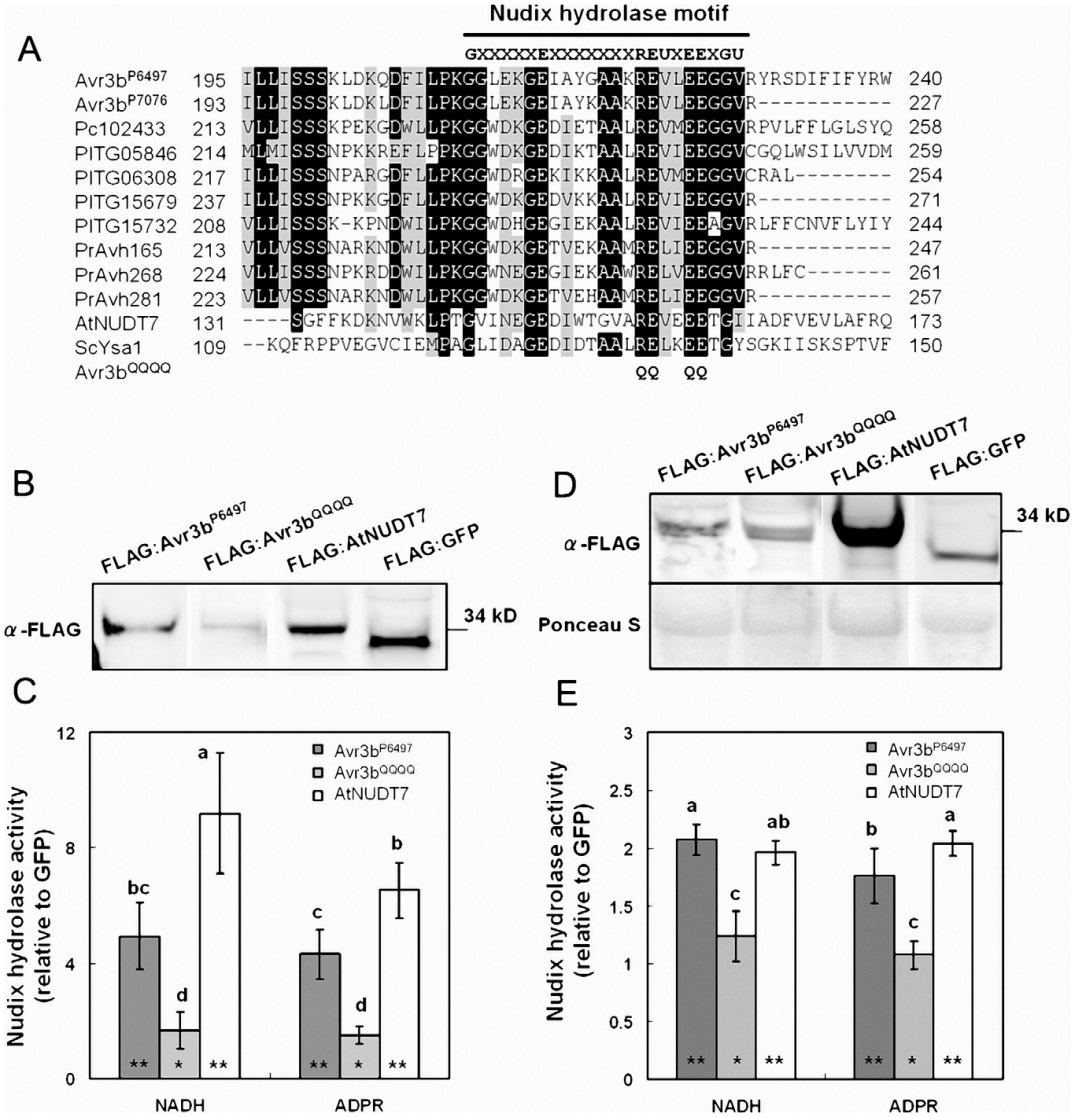
Avr3b is an ADP-ribose/NADH pyrophosphorylase. (**A**) Sequence alignment of the Nudix motif region from Avr3b homologs and characterized nudix hydrolases. Pc102433 is a predicted *P. capsici* RXLR effector. PITG05846, PITG06308, PITG15679, and PITG15732 are predicted *P. infestans* RXLR effectors. PrAvh165, PrAvh268, and PrAvh281 are *P. ramorum* RXLR effectors. AtNUDT7 is an ADP-ribose/NADH pyrophosphorylase from *Arabidopsis.* ScYSA1 is an ADP-ribose pyrophosphorylase from *Saccharomyces cerevisiae.* The black line refers to the Nudix hydrolase motif “G5XE7XREUXEEXGU.” The threshold for amino acid shading in this alignment is 70%. Avr3b^QQQQ^ is an Avr3b^P6497^ non-functional mutant; the glutamine (Q) substitutions in the nudix hydrolase motif are marked. (**B**) Western blot of purified Avr3b and control proteins. Immuno-precipitated recombinant proteins FLAG:AtNUDT7, FLAG:GFP, FLAG:Avr3b^P6497^ and predicted non-functional Avr3b mutant FLAG:Avr3b^QQQQ^ were detected with anti-FLAG antibody. (**C**) Enzyme assays of purified Avr3b and control proteins. The hydrolase activities of FLAG:AtNUDT7, FLAG:Avr3b^P6497^ and FLAG:Avr3b^QQQQ^ were measured separately with FLAG:GFP as a control for each assay. The relative hydrolase activity was calculated as a ratio of Avr3b/AtNUDT7 over GFP. ADPR: adenosine diphosphate ribose, NADH: nicotinamide adenine dinucleotide (reduced form). Bars represent standard errors from 6 independent replicates each. Student's t-test was used to compare whether testing sample activities are significantly different from the GFP control. The double asterisk and single asterisk indicates statistical significance p <0.01 and p <0.05, respectively. The letters represent statistical significance (P <0.01) as measured by Duncan's multiple range test. (**D**) Western blot of the total plant extract from the *Agrobacterium* infiltrated region. The supernatants from FLAG:AtNUDT7, FLAG:GFP, FLAG:Avr3b^P6497^ and FLAG:Avr3b^QQQQ^ transgenic *N. benthamiana* leaves were electrophoresed and detected with anti-FLAG antibody. (**E**) Enzyme assays of the total protein extracts from the *Agrobacterium* infiltrated region. The relative hydrolase activities were measured as a ratio to extracts from a GFP-expression control. Bars represent standard errors from 4 independent replicates each. Student's t-test was used to compare whether testing sample activities are significantly different from the GFP control. The double asterisk and single asterisk indicates statistical significance p <0.01 and p <0.05, respectively. The letters represent statistical significance (P <0.01) as measured by Duncan's multiple range test.

Sequence alignment of Avr3b family members plus two characterized Nudix hydrolases (AtNUDT7 from *Arabidopsis* and ScYSA1 from *Saccharomyces cerevisiae*) revealed that all of these sequences contain the conserved residues of the Nudix hydrolase motif (GX5EX7REUXEEXGU) (Figure 4A). The Nudix motif is located at the C-terminus of each of the oomycete effectors. The motif is located at the extreme C-terminus in many family members including *P. capsici* Pc102433, *P. infestans* PITG05846, PITG06308, PITG15679, and the three *P. ramorum* Nudix hydrolase effectors, a pattern similar to that of Avr3b^P7076^ (Figure S3). However, in Avr3b^P6497^ and PITG15732 the motif is closer to the middle of the protein, which is more similar to AtNUDT7 and ScYSA1.

### Avr3b has ADP-ribose/NADH pyrophosphorylase activity

To examine whether Avr3b has Nudix hydrolase activity, and to identify possible substrates for it, we expressed FLAG:Avr3b^P6497^ (with the FLAG tag fused to the Avr3b N-terminus) in *N. benthamiana* (attempts to produce active protein in *E. coli* were unsuccessful). Western blot data showed a clear 34∼35 kDa band which suggested the recombinant protein was expressed. As a positive control, we fused AtNUDT7 from *Arabidopsis* with a FLAG tag. We then conducted immuno-precipitation using anti-FLAG M2 affinity gel. After immuno-precipitation, western blotting of the elution samples showed clear signals, indicating that, Avr3b protein had been recovered (Figure 4B). To test the hydrolase activity of the precipitated FLAG:Avr3b^P6497^ protein and its possible substrates, relative hydrolase activity was measured as a ratio of FLAG:Avr3b^P6497^ samples over immuno-precipitated GFP, using a series of nucleotide derivatives as substrates. The results showed that FLAG:AtNUDT7 and FLAG:Avr3b^P6497^ immunoprecipitates could both hydrolyze ADPR and NADH, as shown in Figure 4C and Table S5. The AtNUDT7 showed higher activity than Avr3b^P6497^. To confirm Avr3b^P6497^ that is a nudix hydrolase, and to validate that the hydrolase activity in the immunoprecipitates was due to Avr3b, we mutated Avr3b^P6497^ at four conserved residues (R220Q, E221Q, E225Q, E226Q simultaneously, Figure 4A) to generate a non-functional Nudix mutant [39], called Avr3b^QQQQ^. The hydrolase activity of immunoprecipitated Avr3b^QQQQ^ was significantly weaker than Avr3b^P6497^, on either ADPR or NADH, and not significantly greater than the GFP control (Figure 4C). Besides ADPR and NADH, other nucleotide derivatives including NADPH, NAD, Ap4A, FAD and Coenzyme A were also examined as possible substrates for Avr3b^P6497^. The Avr3b^P6497^ immunoprecipitate caused weak but significant hydrolysis of all these derivatives except NAD, whereas there was no significant hydrolysis by the Avr3b^QQQQ^ immunoprecipitate, except possibly of NADPH (Table S5). In our assay, the hydrolase data from AtNUDT7 (Figure 4C), a positive control, is consistent with previous reports [40]. Together, these data are consistent with the hypothesis that Avr3b^P6497^ contains ADP-ribose and NADH pyrophosphorylase activity.

To measure the elevation of total nudix hydrolase activity in the plant tissue resulting from Avr3b^P6497^ expression, we examined the hydrolase activity of total plant extracts. Avr3b^P6497^, Avr3b^QQQQ^ , GFP and AtNUDT7 were transiently expressed in *N. benthamiana* by using *Agrobacterium* infiltration. Western blotting of total plant extracts confirmed expression of the proteins (Figure 4D). Consistent with the results from the immunoprecipitated protein, the extracts from Avr3b^P6497^-expressing plant tissue showed significantly elevated hydrolase activity with NADH and ADPR than the control whereas expression of Avr3b^QQQQ^ did not significantly elevate the hydrolase activity (Figure 4E). No significant elevation of hydrolase activity against NADPH, NAD, Ap4A, FAD or Coenzyme A could be detected in total extracts from Avr3b^P6497^- and Avr3b^QQQQ^-expressing tissue (Table S5). Thus, *in planta* expression of Avr3b^P6497^ elevated the ADP-ribose/NADH pyrophosphorylase activity in the plant tissue by approximately two-fold.

### Avr3b virulence functions depend on its nudix motif, but its avirulence activity does not

**Figure 5:**
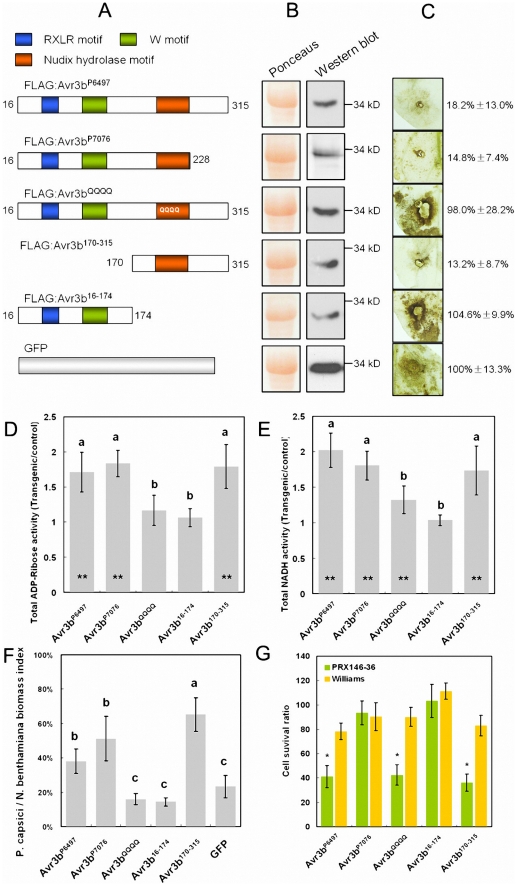
Functional assays of Avr3b alleles and mutants. (**A**) Schematic view of pGR107-FLAG:Avr3b constructs. Avr3b^16-174^, Avr3b^170-315^ and Avr3b^QQQQ^ are derived from the Avr3b^P6497^ avirulence allele. (**B**) Western blot confirmation of *in planta* expression of Avr3b alleles and mutants (as in Figure 4A). (**C**) ROS accumulation assay. DAB staining of inoculation sites around *P. capsici* infection sites on *Agrobacterium*-infiltrated *N. benthamiana* leaf tissue expressing Avr3b alleles and mutants at 36 hpi, as in Figure 3D. The quantification of the DAB staining based on three replicates is shown on the right. The quantification data was shown in a pattern of mean ± standard deviation. (**D,** **E**) ADP-ribose (**D**) and NADH (**E**) hydrolase activity of total protein extracts from *Agrobacterium*-infiltrated *N. benthamiana* tissue transiently expressing Avr3b alleles and mutants, as in Figure 4C. The letters represent statistical significance (P <0.01) determined by Duncan's multiple range test. Bars represent standard errors from 5 independent replicates each. Student's t-test was used to compare whether testing sample activities are significantly different from the GFP control. The double asterisk indicates statistical significance p <0.01 and p <0.05, respectively. (**F**) Virulence assay. The *P. capsici* biomass index in *N. benthamiana* infected tissues was determined by PCR of plant and pathogen DNA as in Figure 3B. The letters represent statistical significance (P <0.01). (**G**) Avirulence assay. Avr3b alleles and mutants were transiently expressed in leaves of soybean cultivar Williams (rps) and PRX146–36 (Rps3b) by double-barreled bombardment, as described in the Methods. Statistical analysis was performed using Wilcoxon rank sum test for each pair of Williams and PRX146–36 measurements. The asterisks indicate a statistically significant difference between Williams and PRX146–36 (P <0.01). A significantly reduced cell survival ratio indicates avirulence activity.

To examine the relationship between Avr3b's Nudix motif and its virulence and avirulence activities, we fused a FLAG tag to the N-terminus of Avr3b^P6497^, Avr3b^QQQQ^, Avr3b^P7076^ (virulence allele), Avr3b^16-174^ (N-terminus of Avr3b^P6497^), and Avr3b^170-315^ (C-terminal of Avr3b^P6497^) (Figure 5A). These proteins were then transiently expressed in *N. benthamiana* leaves using agroinfiltration. Western blots confirmed that all of the proteins were expressed *in planta* with the expected sizes (Figure 5B).

Next, *N. benthamiana* leaves expressing each protein were challenged with *P. capsici* and then stained with DAB to test for suppression of ROS production. Expression of *Avr3b* ^P6497^, *Avr3b* ^P7076^, and Avr3b^170-315^ all noticeably reduced ROS generation, whereas, expression of Avr3b^16-174^ and Avr3b^QQQQ^ failed to do so, similar to expression of the GFP control (Figure 5C).

To measure the elevation in hydrolase activity caused by expression of the Avr3b proteins, total proteins from leaves expressing each construct were assayed with ADP-ribose and NADH as substrates. Protein extracts from leaf tissue expressing Avr3b^P6497^, Avr3b^P7076^, and Avr3b^170-315^ exhibited significantly elevated ADP-ribose and NADH pyrophosphorylase activity whereas leaves expressing Avr3b^16-174^ did not (Figure 5D, 5E). Avr3b^QQQQ^ expression resulted in no significant elevation in ADP-ribose pyrophosphorylase activity, and a weak but not statistically significant elevation in NADH pyrophosphorylase activity (Figure 5D, 5E).

To determine the effect of each mutant on susceptibility to *P. capsici* infection, real-time PCR was used to quantify the pathogen biomass in infected plant tissues expressing each construct. The pathogen biomass was significantly higher in tissues expressing Avr3b^P6497^, Avr3b^P7076^, and Avr3b^170-315^ (p <0.01) than in tissues expressing the GFP control, but was not significantly higher in tissues expressing Avr3b^16-174^ or Avr3b^QQQQ^ (p >0.05) (Figure 5F).

To quantitate the avirulence activity of each Avr3b allele and mutant, the double-barreled version of the co-bombardment assay was used. This assay allows precise quantitation of cell death triggered by a construct by comparing the number of blue spots on one side of a leaf with the number produced by a parallel empty vector bombardment on the other side of the leaf [29]. Consistent with the qualitative assay results shown in Figures 1D and S1, Avr3b^P6497^, but not Avr3b^P7076^, significantly reduced the cell survival ratio to around 40% on leaves containing *Rps*3b, whereas no significant effector-triggered cell death was observed in control leaves lacking *Rps*3b (Figure 5G). Transient expression of Avr3b^QQQQ^ and Avr3b^170-315^ also reduced the cell survival ratio to around 40% in leaves with *Rps*3b but not in those without *Rps*3b. On the other hand, Avr3b^16-174^ did not trigger significant *Rps*3b-dependent cell death (Figure 5G). Therefore the avirulence activity of Avr3b (i.e. triggering of *Rps*3b-dependent cell death) required the C-terminal half of the protein, containing the Nudix motif, but did not require the Nudix motif to be intact.

### Transient silencing of *Avr3b* ^P6497^ impaired *P. sojae* virulence on susceptible soybean

**Figure 6:**
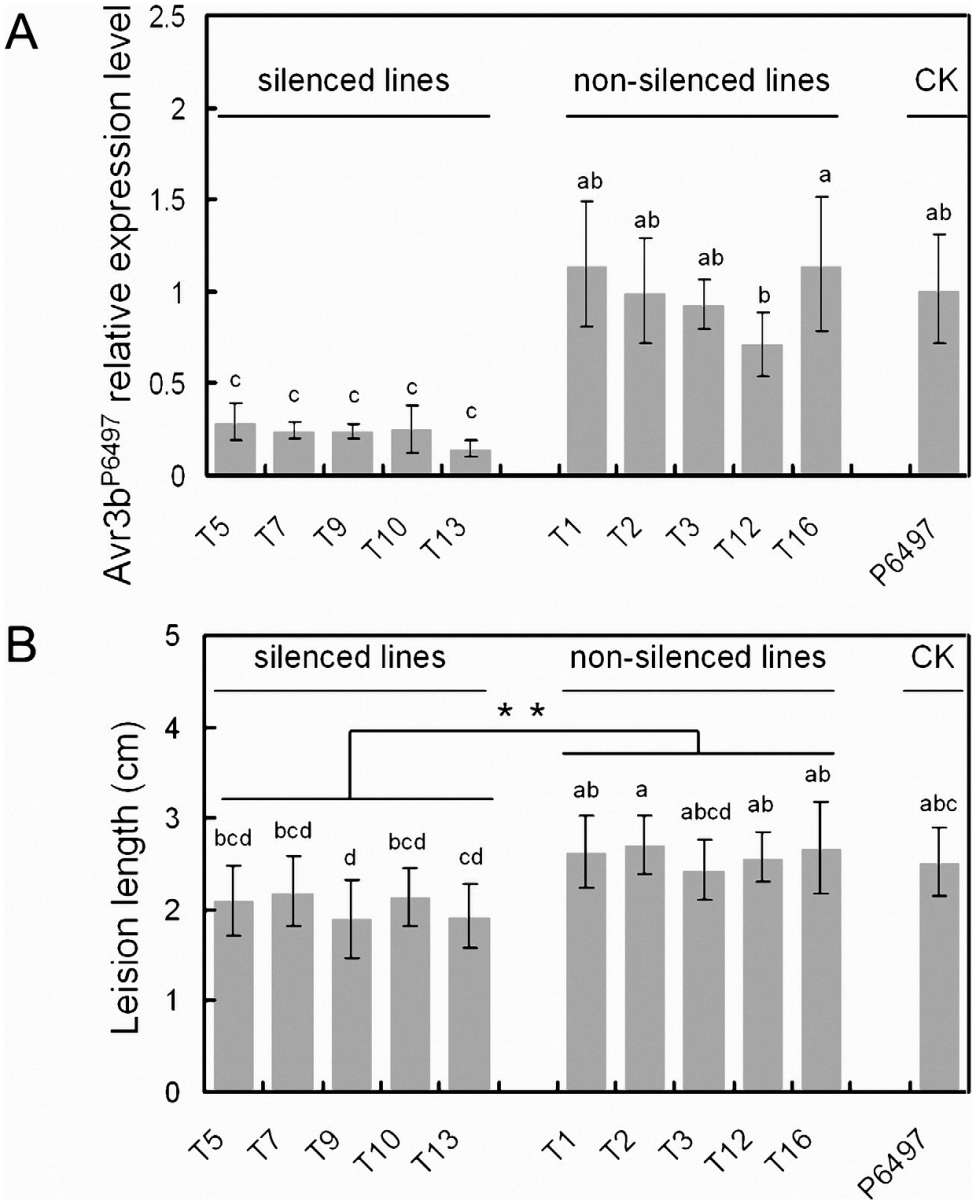
Transient silencing of Avr3b impaired *P. sojae* virulence on soybean. *In vitro* synthesized Avr3b dsRNA was introduced into *P. sojae* P6497, generating 16 lines. Avr3b transient transformants and recipient strain P6497 were examined for Avr3b transcript levels and virulence. RNA samples were extracted at the germinating cyst stage. (**A**) Avr3b transcript levels were determined by Real-time PCR using *P. sojae* actin transcripts as a reference and then normalized to the wildtype (P6497). Bars represent standard errors from 2 independent RNA isolation and Real-time PCR replicates. Letters indicated above each column represent statistical significance based on Duncan's multiple range test (p <0.01). (**B**) The disease lesion lengths in etiolated soybean hypocotyls (susceptible cultivar Williams) infected with zoospores of Avr3b transient transformants and P6497 at 36 hpi. Means and standard errors from at least 10 measurements are shown. Two statistical tests was used. The letters indicated above each column represent statistical significance based on Duncan's multiple range test (p <0.01). The asterisk indicates statistical significance based on student's t-test (p <0.01).

To more directly test the contribution of Avr3b, to *P. sojae* virulence, transient silencing of Avr3b expression in *P. sojae* was carried out. *In vitro* synthesized *Avr3b^P6497^* dsRNA was introduced into *P. sojae* P6497, and 16 lines were recovered. *Avr3b* transcript levels were determined in germinating cysts from two biological replicates of each line using Real-Time PCR. In five lines (T5, T7, T9, T10, T13) levels of the *Avr3b^P6497^* transcript were significantly reduced (14%∼29%) compared to P6497 (Figure 6A). In five non-silenced lines (T1, T2, T3, T12, T16) levels of the *Avr3b^P6497^* transcript were consistently similar to P6497. In six lines, Avr3b transcript levels were not consistent between the two biological replicates; these lines were not examined further.

Zoospore inoculation of etiolated soybean hypocotyls was performed on susceptible soybean cultivar Williams. The disease lesion length was measured to quantify the virulence of *Avr3b*-silenced lines at 36 hpi, as shown in Figure 6B. The lesion lengths generated by the Avr3b-silenced lines were significantly smaller than those of the non-silenced lines and of P6497 (P <0.01). Thus, the silencing of *Avr3b* expression in P6497 significantly impaired virulence.

### The nudix motif is required for Avr3b^P6497^ to suppress ETI

**Figure 7:**
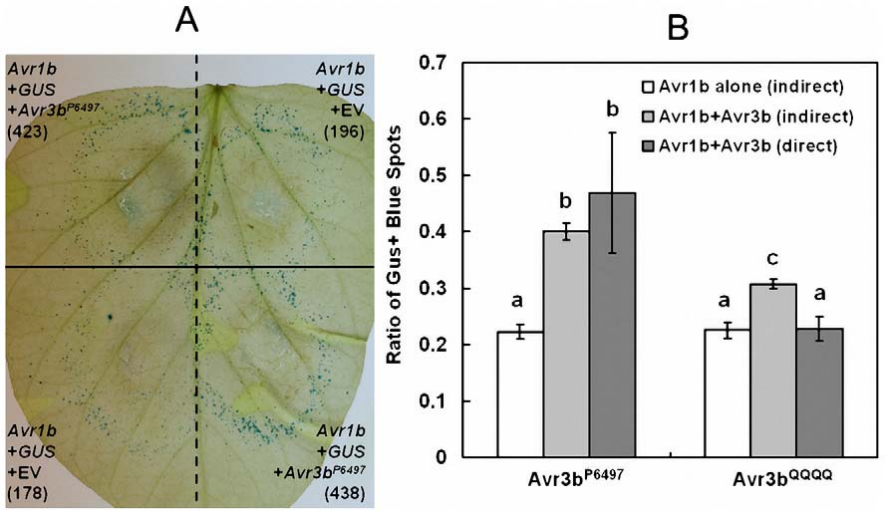
Avr3b suppresses ETI triggered by *P. sojae* effector Avr1b. Suppression of Avr1b-triggered PCD in soybean cultivar L77–1863 (Rps1b) by Avr3b^P6497^ and Avr3b^QQQQ^ measured by indirect and direct double barrel particle bombardment assays. (**A**) Side-by-side comparison of cell death triggered by co-bombardment of Avr1b alone compared to Avr1b+Avr3b^P6497^ on Rps1b soybean. Numbers in parenthesis indicate total GUS positive spots counted for each treatment on the leaf shown. Co-bombardments occur horizontally and are separated by dashed lines. Replicate bombardments are separated by the solid black line. (**B**) Quantitation of suppression by indirect and direct co-bomardment assays. For the indirect assay the number of GUS-expressing spots surviving in the presence of Avr1b on Rps1b soybean, was measured relative to a parallel GUS-only control in the presence (light grey bars) or absence (white bars) of Avr3b^P6497^ or Avr3b^QQQQ^. For the direct assay (dark gray bars) one barrel delivered DNA encoding Avr1b + Avr3b^P6497^ or Avr3b^QQQQ^ and the other barrel delivered Avr1b DNA only. The direct ratio was then multiplied by the cell survival in the presence of Avr1b alone on Rps1b Soybean (white bars) to enable comparison to the results of the indirect assays. Based on the Wilcoxon signed ranks test (direct assays) or the Wilcoxon rank sum test (indirect assays and comparisons of two direct assays), outcomes that were significantly different p <0.001 are marked with different letters.

To further elucidate the virulence mechanism of Avr3b, we tested if Avr3b could suppress ETI in soybean using a double-barreled bombardment protocol [31]. This experiment involves measuring ETI-associated cell death triggered by *P. sojae* effector Avr1b in the presence of soybean *R* gene Rps1b, in the presence or absence of Avr3b. The side-by-side comparison of cell death triggered by co-bombardment of Avr1b alone compared to Avr1b+Avr3b^P6497^ on Rps1b soybean indicated that Avr1b-triggered cell death was strongly suppressed in the present of Avr3b^P6497^ as shown in Figure 7A.

To quantify suppression, and to determine whether the Nudix motif is required for suppression, an indirect and a direct assay were carried out (Figure 7B). For the indirect assay, the number of GUS-expressing spots surviving in the presence of Avr1b on Rps1b soybean was measured relative to a parallel GUS-only control in the presence or absence of Avr3b^P6497^ or Avr3b^QQQQ^. For the direct assay one barrel delivered DNA encoding Avr1b + Avr3b (Avr3b^P6497^ or Avr3b^QQQQ^) and the other barrel delivered DNA encoding Avr1b alone. The results of both the direct and the indirect assays showed that the survival of GUS-spots from Avr1b + Avr3b^P6497^ was significantly higher than from Avr1b alone, confirming that Avr3b could suppress Avr1b-triggered cell death. The survival of GUS-spots from Avr1b + Avr3b^QQQQ^ was significantly less than Avr1b + Avr3b^P6497^ and not significantly higher than from Avr1b alone indicating that Avr3b^P6497^ suppression of Avr1b mediated cell death is dependent on the nudix motif activity.

## Discussion

Oomycete and fungal avirulence effectors form a highly diverse class of proteins, with few if any common sequence signatures. Therefore, the cloning of most oomycete and fungal Avr effectors has relied heavily on genetic mapping and/or genomic subtraction techniques. The discovery of the RXLR host-targeting signal motif in oomycetes has provided a rapid way to identify new Avr effector candidates in conjunction with mapping or screening strategies. For instance, RXLR effector prediction and high-throughput functional screening aided in the discovery of the *P. infestans* genes *Avr3a*, *Avr-blb1*, and *Avr-blb2* [11], [16]. By combining RXLR effector prediction with transcriptional patterns, two RXLR effectors encoded by the *P. sojae* avirulence genes *Avr3a* and *Avr3c* were identified [12], [14]. Here, in addition to RXLR effector prediction and transcriptional data, sequence polymorphism analysis [41] was also used for the discovery of the *P. sojae* Avr effector Avr3b. This powerful combination of methods could significantly accelerate the identification of additional oomycete and fungi avirulence effectors.

The avirulence allele of *Avr3b, Avr3b* ^P6497^, encodes a protein with an intact RXLR motif, a W-motif [5], [30], a Nudix hydrolase motif and two cysteine residues. In the virulence allele, *Avr3b* ^P7076^, a premature stop codon creates a 90-amino-acid C-terminal deletion that removes one cysteine and places the Nudix motif at the extreme C-terminus of the protein. In the non-deleted region, sequence variation results in 46 amino acid substitutions and two deletions, making Avr3b the most polymorphic Avr effector among all reported *P. sojae* Avr genes [12], [13], [14], [15]. Transcripts from *Avr3b* ^P7076^ accumulate at a significantly lower level than from *Avr3b* ^P6497^. However, it is unlikely that *Avr3b* ^P7076^ is a pseudogene because transcripts for this gene are detectable and *in planta* transient expression of Avr3b^P7076^ resulted in pyrophosphorylase enzyme activity and suppression of plant immunity.

Most RXLR effectors in *Phytophthora* have diverged in sequence to such a degree that the identification of orthologous effectors across species is often difficult or impossible. This is likely a result of the rapid diversification of RXLR effectors caused by the evolutionary host-pathogen arms race [3], [6], [18], [23], [42]. However, *P. sojae* Avr3b has one ortholog in *P. capsici*, three in *P. ramorum*, and four in *P. infestans*, forming an Avr3b-like Nudix RXLR effector family. Sequence alignment of the Avr3b family members shows that most of the sequence similarity occurs in the C-terminal region of the Nudix hydrolase motif (Figure S3). The key residues for Nudix hydrolase activity are conserved, consistent with enzyme activity being required for normal function. Avr3b from *P. sojae* has two cysteine residues, a feature that is uncommon in intracellular RXLR effectors but is not unusual for apoplastic effectors from oomycetes or fungi. However the positions of the cysteine residues are not conserved in other Avr3b-like effectors. Whether these cysteine residues are required for avirulence or virulence activities needs further investigation. With regard to expression of the Avr3b orthologs, microarray data shows that two *P. infestans* Avr3b-like effectors PITG06308, PITG15679 are up-regulated by 2.5 and 2.2 fold at 2 dpi, compared to mycelium stages, respectively [23], suggesting Avr3b-like effectors might also function during *P. infestans* infection. The orthologs' conservation among *Phytophthora* species suggests that they that provide a common virulence mechanism. However, this remains to be tested.

Recently evidence has shown that *P. sojae*, *P. infestans*, *P. capsici*, *M. oryzae*, *M. lini* and other pathogen effectors can enter inside plant cells, presumably to promote virulence [7], [8], [9], [10], [43], [44]. Discrete targeting motifs have been identified in these effectors that are required for their translocation into host cells [10], [43], [45], [46]. Like previously reported oomycete avirulence effectors [3], [11], [12], [14], [15], [16], [17], [18], [20], Avr3b has a signal peptide leader and an RXLR motif at its N-terminus. In our assays, expression of Avr3b in soybean or *N. benthamiana* cells without its signal peptide resulted in avirulence and virulence activities, suggesting that Avr3b normally acts inside plant cells; thus it should be able to enter into plant cells during infection. The RXLR motif was also conserved in the Avr3b-like Nudix RXLR effector family. Except for PITG15732 from *P. infestan*s (RFLR) and PrAvh268 from *P. ramorum* (RSLH), all the other Avr3b-like Nudix RXLR effectors have the sequence “RSLR” (Figure S3). The dEER motif that is associated with the RXLR motif was not as highly conserved as the RXLR motif in the Avr3b family.

We failed to identify any Avr3b-like Nudix RXLR effectors in the genome of *H. arabidopsidis*, which has a reduced set of RXLR effectors [22] or in the genome of *P. ultimum,* which has no other RXLR effector genes [47]. Sequencing of additional oomycete pathogen genomes will be required to confirm whether this family is only present in *Phytophthora* species.

Nudix hydrolases are a family of pyrophosphatases containing the highly conserved Nudix motif GX5EX7REUXEEXGU. The family is widely distributed in many organisms, including viruses, bacteria, Archaea, and eukaryotes [39]. Nudix hydrolases catalyze the hydrolysis of a variety of nucleoside diphosphate derivatives linked to a second moiety with varying degrees of specificity [48]. The substrates of Nudix hydrolases identified so far include di- and triphosphates and their oxidized forms, dinucleoside polyphosphates, nucleotide sugars, NADH, coenzyme A, and the mRNA cap [39], [48]. In the genome of the model plant *Arabidopsis*, 29 putative Nudix hydrolases have been identified, and the substrates of a few *Arabidopsis* Nudix hydrolases have been characterized [49]. Like Avr3b, the *Arabidopsis* Nudix hydrolases AtNUDT2, AtNUDT7, and AtNUDT6 use both ADP-ribose and NADH as substrates [40], [50]. Interestingly, among these hydrolases, *AtNUDT*7 was reported to be a pathogen-responsive gene whose induction depends on plant defense regulatory genes *EDS*1 and *PAD*4 [51]. Furthermore, *AtNUDT*7 knockout mutants showed enhanced resistance against *Pseudomonas syringae* and *H. arabidopsidis*, suggesting that ADP-ribose/NADH pyrophosphatases may act as negative regulators of plant immunity to pathogenic bacteria and oomycetes [51]. Therefore, it is reasonable to suppose that pathogen effectors like Avr3b might mimic negative regulators of plant immunity such as AtNUDT 7 to repress plant defense.

Recently, a few putative type-three secretion system (TTSS) effectors with the Nudix motif were identified from the plant pathogenic bacterium *Ralstonia solanacearum* [52], suggesting that this pathogen might also translocate Nudix proteins into plant cells as virulence factors. However, those TTSS effectors were not tested for enzyme activity nor was their contribution to virulence measured. Recombinant Avr3b expressed *in planta* elevated NADH and ADP-ribose pyrophosphatase activities in plant extracts, converting NADH to a reduced form of nicotinamide mononucleotide (NMNH) plus AMP, and ADP-ribose to AMP plus ribose 5-P. The NADH and ADP-ribose pyrophosphatase activity co-purified with Avr3b protein in immunoprecipitation experiments, and the activity was not present when the nudix motif mutant Avr3b^QQQQ^ was expressed. Therefore, it is very likely that Avr3b protein has NADH and ADP-ribose pyrophosphatase activity. However, because Avr3b was not purified to homogeneity, we cannot absolutely rule out an indirect stimulation and co-purification of a plant Nudix hydrolase with wildtype Avr3b in these experiments. NADH/NAD+ turnover plays an important role in maintaining the ROS balance. Recent reports have shown that AtNUDT7 and AtNUDT2 modulate redox homeostasis in response to biotic or abiotic stress by nucleotide recycling from free ADP-ribose molecules [40], [53], [54], [55]. Thus, we hypothesize that inside plant cells Avr3b can suppress pathogen-triggered ROS accumulation by reducing NADH content and recycling nucleotides from ADP-ribose. However, these mechanisms remain to be further investigated.

Rps3b recognizes the presence of Avr3b through the Avr3b C-terminal region (170 to 315 aa) but not the N-terminal region (16 to 174 aa) (Figure 5G). This is consistent with previous reports indicating that the C-terminal sequences of most *P. sojae, P. infestans* and *H. arabidopsidis* avirulence effectors show signs of positive selection [11], [30], [31], [56]. In contrast to other avirulence effectors, the Avr3b^P6497^ C-terminal region contains an identifiable enzyme domain, a Nudix hydrolase domain. In this study, we generated a mutant version of the protein, Avr3b^QQQQ^, that apparently lacks Nudix enzyme activity but nonetheless could still trigger a defense response in the presence of Rps3b. This result suggests that Rps3b recognition might not depend on Nudix hydrolase activity. However, Avr3b^QQQQ^ retains residual hydrolase activity. Thus we can not rule out the possibility that Avr3b avirulence activity also required Nudix activity because the threshold for Avr3b-mediated avirulence activity is likely to be much lower than for its virulence activity. Due to the numerous polymorphic sites present within the Avr3b^170-315^ domain, the particular residues responsible for Avr3b-Rps3b recognition cannot readily be discerned. Only Avr3b mutants retaining Avr3b ADP-ribose and NADH pyrophosphorylase activity could suppress ETI and increase *Phytophthora* biomass in infected plant tissue, suggesting that the enzymatic activity is required to promote *Phytophthora* virulence.

*Phytophthora* genomes encoded hundreds of RXLR effector genes, suggesting many of these effectors might be redundant in function [57]. For examine, either overexpression or silencing of *P. sojae* Avr gene *Avr3a/5* does not significantly effect *P. sojae* virulence on susceptible soybean [19]. Overexpression of *Avr1b* in *P. sojae* makes transformants more aggressive on soybean [30], but the effector is naturally silenced in some isolates [13]. On the other hand silencing of *Avr3a* impaired *P. infestans* pathogenicity on *N. benthamiana* [33], and silencing of Avh172 and Avh238 in *P. sojae* also impaired virulence [31], suggesting that these effectors are essential virulence factors. In this paper, transient silencing of *Avr3b* ^P6497^ compromised the virulence of transformant recipient strains on susceptible soybean cultivar, identifying Avr3b also as an essential virulence factor. This is consistent with our finding that both Avr3b virulence and avirulence alleles are transcribed and both proteins retain Nudix enzyme activity. In conclusion, Avr3b plays dual roles (avirulence and virulence) in the *P. sojae* - soybean interaction. The virulence allele Avr3b^P7076^ may be considered to be a compromise between host recognition pressures and pathogen fitness.

## Materials and Methods

### Plant and microbial culture, and virulence scoring

Detailed information about the *P. sojae* strains used in this study is listed in Table S6. The *P. capsici* (Pc263) and *P. parasitica* (P24-3) strains were obtained from the *Phytophthora* species collection at Nanjing Agricultural University. All of these isolates and the P6497× P7076 F_1_ progeny and F_2_ progeny used in this paper were routinely maintained on 2.5% vegetable (V8) juice medium at 25°C in the dark [58]. *Phytophthora sojae* mycelia, zoospores, and cysts were prepared as previously described [59]. Cysts germinating at 25°C for 6 hours were used for this study. For RNA samples, infection assays with *P. sojae* were performed by sandwiching *P. sojae* mycelium between pairs of soybean leaves [60]. The mycelium was removed at 6 hpi, and the infected soybean leaves were collected at 12 hpi and 24 hpi. All of the collected samples were immediately frozen in liquid nitrogen and stored at −80°C until used for RNA isolation.

Soybean (*Glycine max*) cultivar Williams (*rps*), Williams isoline L88–1479 (*Rps*3b), and PRX146–36 (*Rps*3b) from the collections at Nanjing Agricultural University and Northeast Agricultural University (Harbin, China) were used to score the virulence of *P. sojae* cultures. Williams isoline L77–1863 (*Rps*1b) was from the collection at the Virginia Bioinformatics Institute. Etiolated soybean seedlings were grown in vermiculite soaked with water at 25°C without light for 4 days before harvest for co-bombardment. For light-grown soybeans, ten soybean seeds were sown in 10 cm pots (a minimum of three pots per isolate) containing a soil (70%) and vermiculite (30%) mix soaked with water. Soybeans were grown in a greenhouse with a 16-hour photoperiod at 25°C. Soybeans were grown for 7 days for virulence assays, or for 13 to 14 days for use in co-bombardment. *Phytophthora sojae* cultures were grown on 0.9% (v/v) V8 agar plates 5 to 7 days prior to light-grown plant inoculations. The virulence of *P. sojae* cultures was scored in exactly the same manner as that previously described [14]. A minimum of three independent replicates of the disease assay were performed for each *P. sojae* culture tested. *N. benthamiana* plants were grown at 25°C with a 16 hr photoperiod in a greenhouse in styrofoam cups containing disinfected soil. Plants of 5 to 6 weeks old were used for agroinfiltration.

### Bioinformatics

In total, 395 predicted RXLR effectors in *P. sojae* P6497 were previously predicted [42]. For DGE expression data, RNA samples were collected at Nanjing Agricultural University, and sequencing and analysis were performed at Beijing Genomics Institute. Affymetrix microarray data were previously reported [12], [31]. All RXLR sequences were used as queries for BLAST searches of the *P. sojae* EST unigene database (http://vmd.vbi.vt.edu) [61] , with an E-value cutoff at e^−20^. We identified a total of 131 RXLR effectors that are expressed in *P. sojae* P6497. *P. sojae* 454 genome sequencing data (P7064, P7074, and P7076) [31] were accessed from Virginia Microbial Database (http://vmd.vbi.vt.edu). Nudix homologs were identified in two ways: genome annotation searching and BLAST searching. The *P. sojae*, *P. capsici* and *P. ramorum* homolog searches were performed at the Joint Genome Institute database (http://genome.jgi-psf.org), the *H. arabidopsidis* search was performed at Virginia Microbial Database, and the *P. infestans* search was conducted at the Broad Institute *P. infestans* database (http://www.broadinstitute.org). For *Pythium ultimum* genome searching, the genome sequence was downloaded from the *Pythium* genome database available at http://pythium.plantbiology.msu.edu and a local BLAST search was conducted. SignalPv3.0 (http://www.cbs.dtu.dk/services/SignalP) was used for secretion signal peptide prediction. Protein domain and motif analyses were conducted using the NCBI conserved domain database (http://www.ncbi.nlm.nih.gov/Structure/cdd/cdd.shtml) and Motif Scan (http://myhits.isb-sib.ch/cgi-bin/motif_scan). Sequence alignment was performed using BioEdit2005 (http://www.mbio.ncsu.edu/bioedit/bioedit.html). The Avr3b-like family member sequences have been deposited into NCBI GenBank with accession numbers (Table S7).

### Generation of F_2_ mapping population and scoring of genotypes

F_1_ hybrids were derived from P6497 (*Avr3b*) × P7064 (*avr3b*) crosses. Oospores from F_1_ hybrids were produced as described [14]. Single germinating oospores were harvested for DNA isolation. DNA isolation was performed using a fast DNA isolation kit (Axygen Biotech, China). Of 79 germinating oospores, three F_1_ hybrids were confirmed using two different CAP DNA markers corresponding to polymorphisms within the predicted genes *Avh*20 and *Avh*238). One of the F_1_ hybrids was cultured on 2.5% (v/v) V8-medium plates for F_2_ progeny oospore generation. A total of 71 F_2_ progeny were produced to establish an expanded mapping population to test Avr3b candidate effectors. For each F_2_ individual, virulence was scored on Williams (*rps*) and L88–1479 (*Rps*3b) plants, and DNA samples from mycelia were also isolated for genotyping. CAP markers for seven Avh genes (*Avh*238, *Avh*307, *Avh*113, *Avh*258, *Avh*20, *Avh*288, and *Avh*6) were designed based on polymorphisms between P6497 and P7076; details are provided in Table S2. The genotype of the candidate gene and the virulence type of the F_2_ progeny were compared. For *Avr3b* allele sequencing in different strains, the specific primers Avh307 MF, Avh307 MR, Avh307 MFa, and Avh307 MFb were used for PCR amplification and sequencing. Primer sequences are presented in Table S2.

### Soybean transient expression assays

The *Avr3b^P6497^* and *Avr3b^P7076^* genes, excluding regions encoding the predicted signal peptide sequences, were amplified using specific primers and cloned into a 35S promoter-derived plant expression vector pFF19 using *Bam*H I and *Sph* I restriction sites (primers sequences are shown in Table S8). Similarly, constructs for bombardment of *Avr3b^P6497^*-derived mutants (Avr3b^16-174^, Avr3b^170-315^ and Avr3b^QQQQ^) were also generated. Co-bombardment and transient expression assays for Avr3b^P6497^ and Avr3b^P7076^ on etiolated soybean hypocotyls were performed as described [37]. Leaves were photographed using a digital camera system (V12, Zeiss Germany). For Avr3b-derived mutant avirulence assay and Avr3b suppression of Avr1b ETI assay, double-barreled particle bombardment assays on soybean leaves were performed as previously described [30]. Both hypocotyl co-bombardment and double-barreled particle bombardment were conducted using a Bio-Rad (USA) He/1000 particle delivery system. For each paired shot, the logarithm of the ratio of the spot numbers of the test construct to that of the control was calculated. The log ratios obtained from the *Rps*3b and non-*Rps*3b leaves were then compared using the Wilcoxon rank-sum test.

### RNA isolation and real-time RT-PCR

To monitor *Avr3b* transcript profiling in *P. sojae* P6497 by real-time RT-PCR, total RNA samples from mycelia, zoospores, cysts, germinating cysts, and infected plant tissue samples were extracted using a PureLink RNA Mini Kit (Invitrogen USA). For Avr3b transformants, germinating cysts was prepared at 9 and 11 days after transformation. The RNA samples were isolated from these germinating cysts (two biological replicates for each transformants). The first-strand cDNA was synthesized using Superscript II reverse transcriptase (Invitrogen) following the manufacturer's directions. For *Avr3b* transcript profiling analysis, SYBR green real-time RT-PCR assays were carried out. Primer pairs (Table S8) were designed at the identical region between Avr3b^P6497^ and Avr3b^P7076^. Two *P. sojae* housekeeping genes were selected as endogenous controls, namely actin (JGI Gene ID: 109046) and the molecular chaperone HSP70 superfamily gene (JGI Gene ID: 144810). PCR reactions (20 µL) included 20 ng cDNA, 0.2 µM of each primer, and 10 µL SYBR Premix ExTaq (TaKaRa Inc., Dalian, China). Reactions were performed on an ABI PRISM 7300 fast real-time PCR system (Applied Biosystems, USA) under the following conditions: 95°C for 30 s, 40 cycles of 95°C for 5 s, and 60°C for 31 s; followed by 95°C for 15 s, 60°C for 1 min, and 95°C for 15 s to obtain melt curves. The expression of each gene relative to average Ct values of the two housekeeping genes (Ct  =  Ct_gene_ - Ct_HKaverage_) was determined and analyzed using ABI 7300 System Sequence Detection Software Version 1.4 [62].

### Construction of recombinant *Agrobacterium* binary PVX vectors

Sequences corresponding to Avr3b^P6497^ and Avr3b^P7076^ with secretion signal peptides replaced by a FLAG tag sequence were amplified from P6497 and P7076 genomic DNA using high-fidelity DNA polymerase (TaKaRa, Inc.) with primers described in Table S8. The PVX vector pGR107 for *N. benthamiana* transient expression assay was isolated using a plasmid spin column small isolation kit (Axygen Biotech. China.). For the Avr3b^P6497^ mutant, a method similar to that used for vector construction was used. The Avr3b^QQQQ^ mutant was generated with additional primers (Table S8) by overlapping PCR. High-fidelity PCR products were sub-cloned into pGR107 pre-digested by the restriction enzyme *Sma* I. Recombinant binary vectors were maintained and propagated in the *Escherichia coli* strain JM109, grown in the presence of 50 µg/mL kanamycin. The recombinant binary vectors were transformed into the *Agrobacterium tumefaciens* strain GV3101 by electroporation. After growing at 28°C on LB agar plates supplemented with 50 µg/mL kanamycin as selective agents for 2 days, individual *Agrobacterium* colonies were verified with PCR using vector primers. *Agrobacterium tumefaciens* was grown in LB broth cultures supplemented with 25 µg/mL kanamycin for 2 day at 28°C with constant shaking. The cultures were centrifuged at 4,000 rpm for 4 min in a tabletop centrifuge. The pellet was resuspended in 1 mL of induction medium (10 mM MES, 10 mM MgCl_2_, and 150 mM acetosyringone, pH  = 5.6). The final concentration of *Agrobacterium* cells was adjusted to OD_600_  = 0.4. Leaves of *N. benthamiana* were infiltrated with *Agrobacterium* cultures using a blunt syringe. The infiltrated plants were then kept in a greenhouse for 72 hours prior to Western blot, immuno-precipitation, or virulence assays.

### Virulence assay of *Phytophthora* on *N. benthamiana*

*Agrobacterium*-infiltrated *N. benthamiana* plants were grown in a greenhouse for 48 hours, and transformed leaves were then detached and maintained on half-strength MS medium in a petri dish. Next, 2.5% V8 juice agar plugs (0.5×0.5 cm) infested with fresh *P. capsici* or *P. parasitica* mycelia were inoculated onto the infiltrated regions. The diameter of the disease lesion was photographed and measured at 36 hpi. Total DNA isolated from *P. capsici*-infected regions (2×2 cm) was isolated at 36 hpi. Real-time PCR was used to quantify the ratio of host to pathogen ratio DNA sequences, employing primers specific for the *N. benthamiana* and *P. capsici* housekeeping actin genes (Table S8). Three independent biological replicates were conducted. Diseased plant tissues at 16 hpi were stained by trypan blue and DAB according as described [63]. Quantification DAB was performed by analyzing DAB staining image by a combination of Photoshop and Quantity One. GFP was considered to be 100% standard.

### Western blot and immuno-precipitation assays

Total plant protein was isolated as described [63] for Western blot and immuno-precipitation assays. The protein samples were quantified using a BioPhotometer (Eppendorf, Germany). A standard sodium dodecyl sulfate polyacrylamide gel electrophoresis (SDS-PAGE) protocol was used for protein separation. The amount of total protein loaded per gel lane varied from 80 µg to 120 µg depending on each experiment. Proteins were transferred onto polyvinylidene difluoride (PVDF) membranes using a semi-wet apparatus (Bio-Rad) according to the product instructions. Western blotting was performed as a standard protocol. Anti-FLAG monoclonal antibody (Sigma-Aldrich) and anti-mouse IgG-peroxidase conjugate (Beyotime Biotech, China) were used as the primary and secondary antibodies. The membrane was treated with Chemiluminescent Peroxidase Substrate-1 (Thermo Scientific Pierce, No. 34080, USA) for 5 min. The membrane was briefly drained and exposed to BioMax (Kodak, USA) light film several times (depending on results) for exposure signal development.

For immuno-precipitation, anti-FLAG M2 affinity gel (Sigma-Aldrich) was used. The gel suspension (40 µL) was transferred into a fresh 1.5 mL test tube and centrifuged in a pre-cooled rotor at 6000 g for 30 s. The resin was washed twice with 1 mL TBS (50 mM Tris HCL, 150 mM NaCl, pH = 7.4), and all washing buffer was removed. Total protein lysate was clarified by centrifugation (10000 g, 2 min) then 1000 µL of the supernatant was added to the washed resin, and the mixture was incubated using a roller shaker for 4 hours at 4°C. The suspension was centrifuged at 6000 g and the supernatant was removed. The remaining resin was washed three times with 1 mL of TBS. A total of 100 µL elution buffer (0.5 M Tris-HCl buffer, 150 ng/µL 3xFLAG, pH = 7.2) was then added to the resin, and the samples were incubated using a roller shaker for 10 min at room temperature. The resin was centrifuged for 30 s at 6000 g, and the supernatants were transferred to fresh tubes previously loaded with 10 µL of 0.5 M Tris HCl and 1.5 M NaCl, pH 7.4. The purified protein samples were used for gel electrophoresis and enzyme activity assay.

### Nudix hydrolase activity assays

A general method for the Nudix hydrolase activity assay was performed to test for Avr3b enzymatic activity [40], [53]; a 50-µL reaction mixture contained 50 mM Tris-HCl, pH 8.5, 5 mM MgCl_2_, 1 mM dithiothreitol, 2 units of calf alkaline phosphatase, 2 mM substrate (ADP-ribose, NADH, FAD, or Ap4A; Sigma-Aldrich), and certain amount of protein samples. Equal amount of protein (5 µg for purified protein, 2 mg for total plant protein extract) was loaded for hydrolase activity assay in each reaction. After incubation for 1 hr at 37°C, the reaction was stopped by adding 150 µL of 1 N H_2_SO_4_. For color development, 100 µL of water and 700 µL of a freshly made mixture containing 600 µL of 3.4 mM ammonium molybdate and 100 µL of 570 mM ascorbic acid was used. The reaction tubes were incubated in a 45°C dry bath for 10 min for color development and then cooled on ice. The solutions were measured using the Du-640 spectrophotometer (Beckman, USA) at A_820_ The reaction without protein was used as a blank control for each substrate. For direct assay of purified proteins, immuno-precipitation eluate was directly added into enzymatic assay reaction mixture.

For hydrolase activity assay of total plant protein extract, two regions per leaf transiently expressing FLAG:GFP and FLAG:Avr3b^P6497^ were created in *N. benthamiana* leaves using *Agrobacterium* infiltration. A total of 0.1 g of GFP- or Avr3b-infiltrated leaf tissue was collected from each leaf. For both kinds of protein preparations, the relative hydrolase enzyme activity was calculated as a ratio of Avr3b extract (A_820_ reading) over GFP extract (A_820_ reading). For NADH and ADPR, 17 and 21 biological replicates were performed, respectively. For FAD, Ap4A, NAD and NADPH, 4 biological replicates were conducted.

### *In vitro* dsRNA synthesis and *P. sojae* transformation

Avr3b *in vitro* dsRNA synthesis was carried out as described [64]. Primers AVH307-T7F/R with T7 promoter sequence added to the 5′ end of both primers (see Table S8) were used to amplify a C-terminal specific Avr3b DNA fragment from the Avr3b^P6497^ allele. The PCR product was cloned into pMD19 vector (TaKaRa) and sequenced. AVH307-T7F/R primers were also used to generate Avr3b *in vitro* dsRNA by using the Megascript RNAi kit (Ambion AM1626). A total of around 200 µg dsRNA was obtained as measured by spectrophotometry. *P. sojae* transient transformation was performed as described [30], [59] with a few modifications [32]. About 100 µg of Avr3b dsRNA was added into 1 mL MMg solution (0.4 M mannitol, 15 mM MgCl_2_, 4 mM MES, pH 5.7) containing 5000–10000 *P. sojae* P6497 protoplasts. After transformation, the regenerated protoplasts were suspended in liquid pea agar (40 °C ) containing 0.5 M mannitol. The visible colonies could be observed after 36 h incubation at 25 °C. A total of 16 single colonies were randomly selected and propagated on V8 agar plates for RNA extraction and virulence assays.

## Supporting Information

Figure S1 **Photographs of soybean leaves after double-barreled co-bombardment, GUS staining, and leaf destaining.** Numbers in parenthesis indicate total GUS positive spots counted for each treatment. Numbering indicates the sequential order of co-bombardments on a given leaf. Co-bombardments occur horizontally and are separated by dashed lines. Different co-bombardments are separated by solid black lines. **(A)** *Avr3b* ^P6497^ **(B)** *Avr3b* ^P7076^ co-bombarded with empty vector control on *Rps*3b soybean. **(C)** *Avr3b* co-bombarded with empty vector control on *rps* soybean.
(TIF)

Figure S2 ***P. parasitica*** **and** ***P. capsici*** **lesion sizes on** ***N. benthamiana*** **plants transiently expressing Avr3b. (A)** *P. parasitica* and *P. capsici* were inoculated onto Avr3b- or GFP-expressing leaves; a photograph was taken at 36 hpi. The white dotted line indicates the disease lesion region. **(B)** The diameter of the disease lesion was measured and the mean and standard error of at least five independent replicates are shown.
(TIF)

Figure S3 **Amino acid sequence alignment of the Avr3b family members.** The threshold for shading is 70%. Blue box indicates RXLR host targeting motif, green box indicates predicted W-motif, orange box indicates Nudix hydrolase motif.
(TIF)

Table S1 **119 expressed** ***P. sojae*** **RXLR effector genes.**
**(DOC)**

Table S2 **Cleaved Amplified Polymorphic (CAP) markers for Avr3b genotyping.**
**(DOC)**

Table S3 **RXLR effectors that are located in the Avh307 region.**
**(DOC)**

Table S4 **Predicted** ***Phytophthora*** **Nudix hydrolase RXLR effectors.**
**(DOC)**

Table S5 **The hydrolytic activities of both pure Avr3b protein and plant protein extract against nucleotide derivatives.**
**(DOC)**

Table S6 **List of** ***P. sojae*** **strains used in this study.**
**(DOC)**

Table S7 **Sequences referred in this paper.**
**(DOC)**

Table S8 **Primers used in this study.**
**(XLS)**
