# Supplementary figures and images for: Phytophthora sojae Avirulence Effector Avr3b is a Secreted NADH and ADP-ribose Pyrophosphorylase that Modulates Plant Immunity
Source: PLoS Pathog. 2011 Nov 10;7(11):e1002353. doi: 10.1371/journal.ppat.1002353 (PMC3213090; doi:10.1371/journal.ppat.1002353)

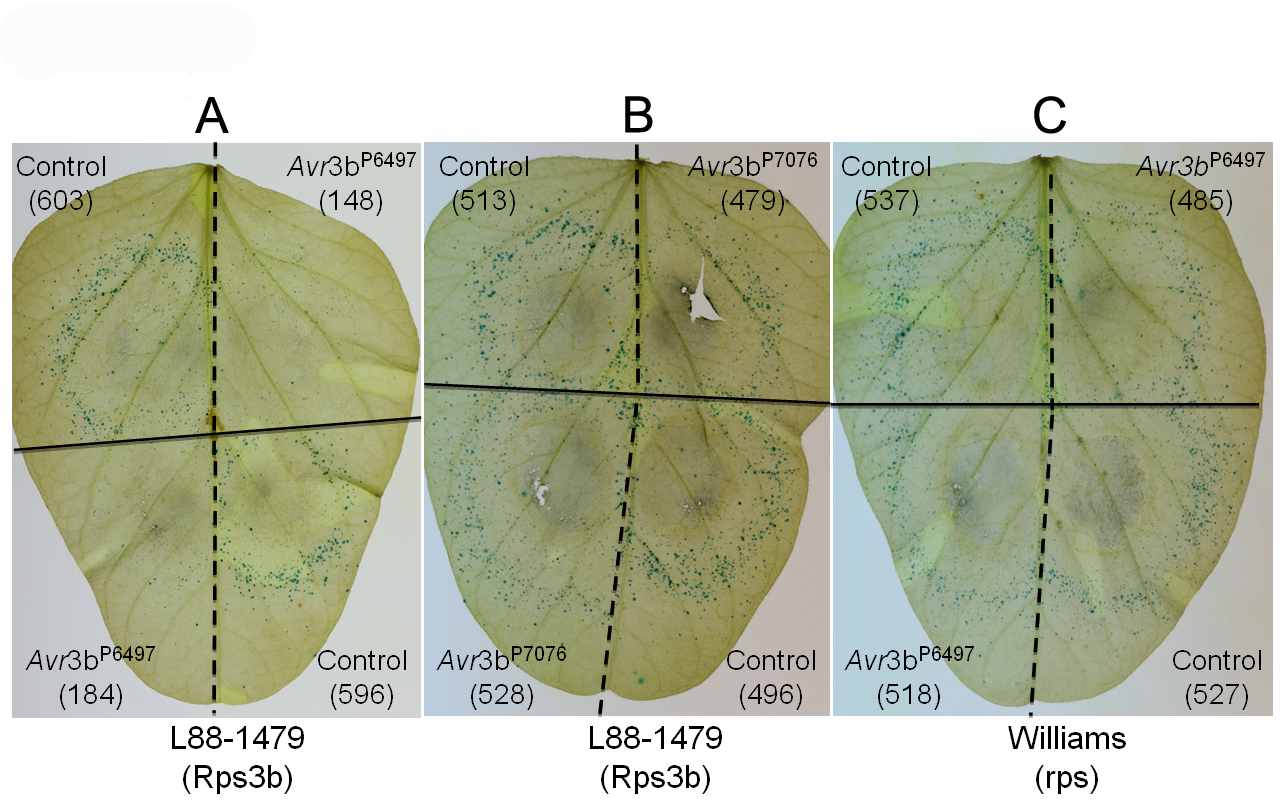

Supplement: Figure S1 — Photographs of soybean leaves after double-barreled co-bombardment, GUS staining, and leaf destaining. Numbers in parenthesis indicate total GUS positive spots counted for each treatment. Numbering indicates the sequential order of co-bombardments on a given leaf. Co-bombardments occur horizontally and are separated by dashed lines. Different co-bombardments are separated by solid black lines. (A) Avr3b P6497 (B) Avr3b P7076 co-bombarded with empty vector control on Rps3b soybean. (C) Avr3b co-bombarded with empty vector control on rps soybean. (TIF) [file ppat.1002353.s001.tif]

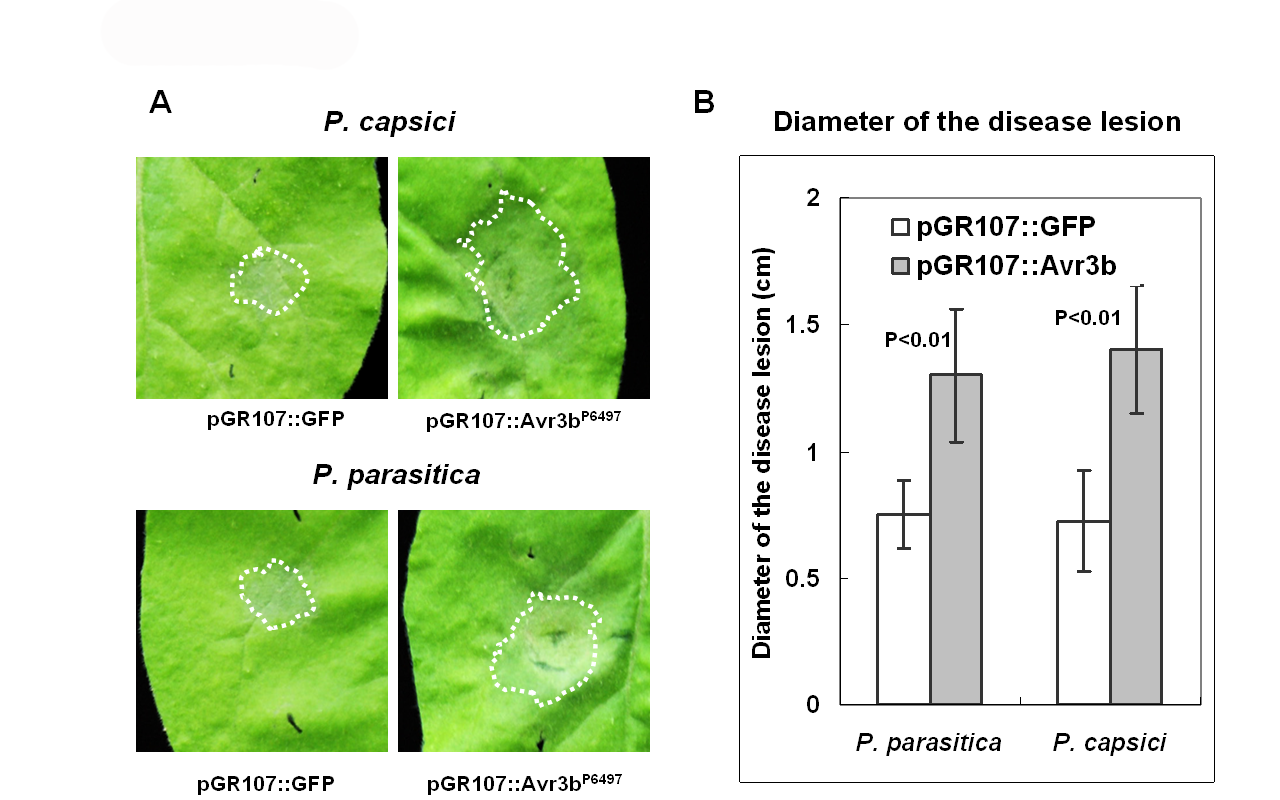

Supplement: Figure S2 — P. parasitica and P. capsici lesion sizes on N. benthamiana plants transiently expressing Avr3b. (A) P. parasitica and P. capsici were inoculated onto Avr3b- or GFP-expressing leaves; a photograph was taken at 36 hpi. The white dotted line indicates the disease lesion region. (B) The diameter of the disease lesion was measured and the mean and standard error of at least five independent replicates are shown. (TIF) [file ppat.1002353.s002.tif]

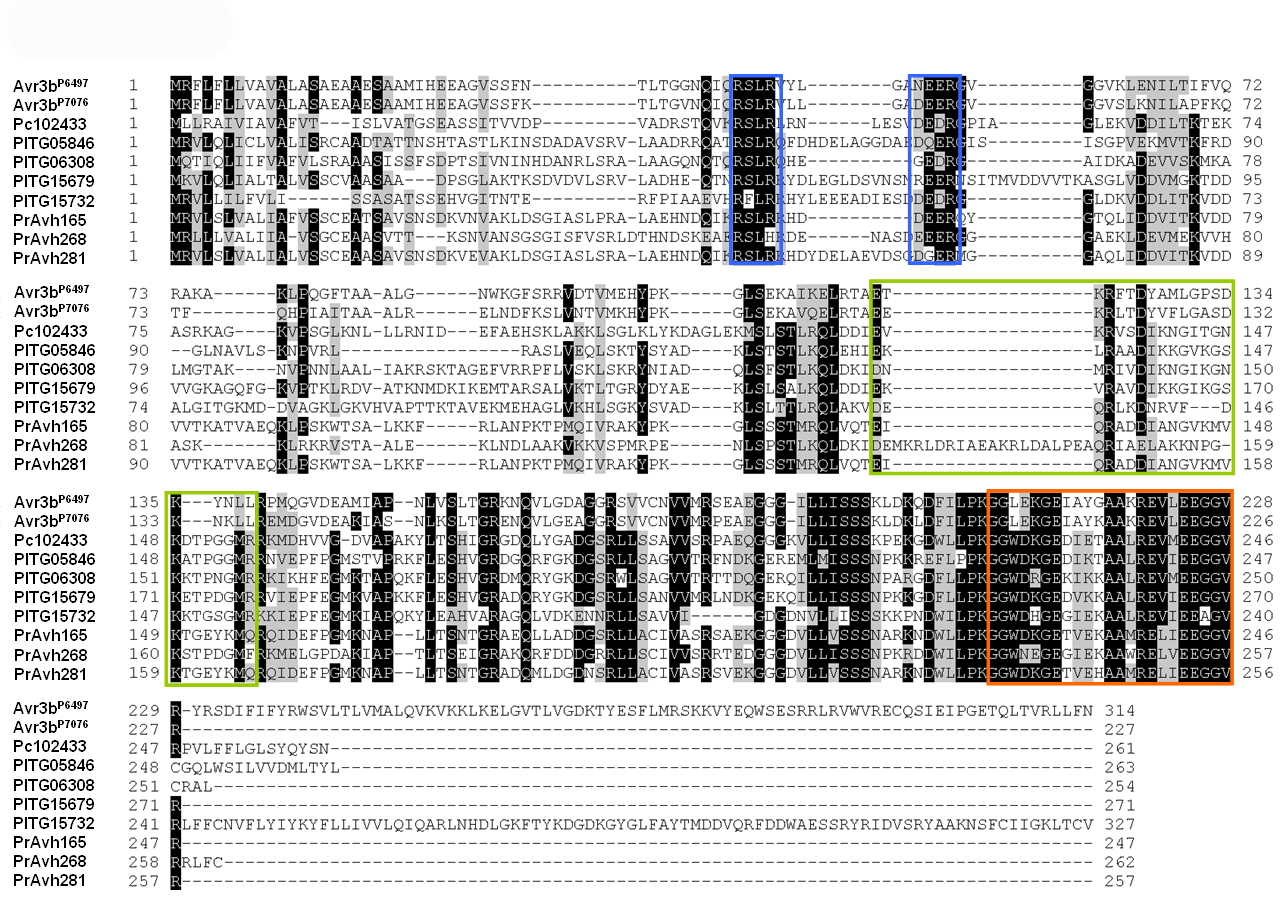

Supplement: Figure S3 — Amino acid sequence alignment of the Avr3b family members. The threshold for shading is 70%. Blue box indicates RXLR host targeting motif, green box indicates predicted W-motif, orange box indicates Nudix hydrolase motif. (TIF) [file ppat.1002353.s003.tif]
