# Supplementary material for: Phytophthora sojae Avirulence Effector Avr3b is a Secreted NADH and ADP-ribose Pyrophosphorylase that Modulates Plant Immunity
Source: PLoS Pathog. 2011 Nov 10;7(11):e1002353. doi: 10.1371/journal.ppat.1002353 (PMC3213090; doi:10.1371/journal.ppat.1002353)
Supplement: Table S1 — 119 expressed P. sojae RXLR effector genes. (DOC) [file ppat.1002353.s004.doc]

**Table S1: 119 expressed *P. sojae* RXLR effector genes**

| **Protein ID^a^** | **Name^b^** | **DGE^c^** | **Affy^c^** | **EST^c^** | **SignalP HMM^d^** | **SP Length^d^** | **RXLR^e^** | **dEER^e^** | **Annotation** |
| --- | --- | --- | --- | --- | --- | --- | --- | --- | --- |
| 158991 | Avh1 |  | + |  | 0.9960 | 21 | RFLR | DAGER | Avr1b^[13]^ |
| 158992 | Avh4 |  | + |  | 0.9980 | 21 | RFLR | DVSSSEER |  |
| 158994 | Avh6 |  | + | + | 0.9980 | 25 | RFLR | DNEER |  |
| 158999 | Avh8 |  | + |  | 1.0000 | 21 | RFLR | DSEEEER |  |
| 159001 | Avh13 |  | + |  | 1.0000 | 21 | RLLR | DEEER |  |
| 159003 | Avh15 | + | + |  | 0.9980 | 23 | RLLR | DVADEER |  |
| 159004 | Avh16 | + | + |  | 1.0000 | 20 | GMLRR | DKINEER |  |
| 159005 | Avh17 | + |  |  | 1.0000 | 24 | RHWR | DEVEEEK |  |
| 159008 | Avh20 |  | + |  | 1.0000 | 21 | RLLR | DNEEER |  |
| 159011 | Avh23 | + | + |  | 0.9970 | 23 | RMLR | DHEER |  |
| 159013 | Avh25 | + | + |  | 1.0000 | 19 | RNLR | DDEDR |  |
| 159015 | Avh27 | + | + |  | 1.0000 | 20 | RFLR | DGSEER | Avr3c^[12]^ |
| 159017 | Avh29 |  | + |  | 1.0000 | 23 | RFLR | DEDR |  |
| 159019 | Avh31 | + |  |  | 0.9850 | 46 | RFLR | DDEEER |  |
| 159025 | Avh38 | + | + | + | 0.9980 | 26 | RSLE | EGEEER |  |
| 159027 | Avh40 |  | + |  | 0.9990 | 20 | RFLR | DDEER |  |
| 159029 | Avh42 | + |  |  | 1.0000 | 21 | RSLR | DEER |  |
| 159037 | Avh52 |  | + | + | 0.9980 | 21 | RLLR | DEQR |  |
| 159038 | Avh53 |  | + |  | 0.9990 | 19 | RLLR | DEER |  |
| 159040 | Avh61 | + | + |  | 0.9910 | 20 | RALR | DATGEER |  |
| 159041 | Avh62 |  |  | + | 1.0000 | 21 | RFLR | DGSEER |  |
| 159043 | Avh64 | + |  |  | 1.0000 | 25 | RFLR | DNEER |  |
| 159046 | Avh67 |  |  |  | 1.0000 | 20 | RRLR | DANEEVEER |  |
| 159050 | Avh73 |  | + |  | 1.0000 | 23 | RALR | DEER |  |
| 159053 | Avh78 | + |  |  | 0.9980 | 27 | RFLR | DGEDEER |  |
| 159055 | Avh80 |  | + |  | 1.0000 | 20 | KRFPR | EHDKR |  |
| 159056 | Avh81 | + | + |  | 0.9990 | 25 | RFLR | ESEGDER |  |
| 159060 | Avh87 | + |  |  | 0.9760 | 31 | RFLR | DEER |  |
| 159064 | Avh92 | + | + |  | 0.9990 | 20 | RLLR | DSPEDR | Avr3a^[14]^ |
| 159065 | Avh94a | + | + | + | 1.0000 | 23 | RLLR | DVEDR |  |
| 159066 | Avh95 | + |  |  | 1.0000 | 23 | RLLR | DVEDR |  |
| 159068 | Avh97 | + |  |  | 1.0000 | 23 | RSLR | DEER |  |
| 159073 | Avh105 | + |  |  | 1.0000 | 21 | RFLR | DSADEER |  |
| 159076 | Avh109 |  | + |  | 0.9980 | 20 | RFLV | KGDR |  |
| 159077 | Avh110 | + | + | + | 1.0000 | 20 | RFLR | DEER |  |
| 159078 | Avh111 | + |  |  | 1.0000 | 20 | RFLR | DLEGEDR |  |
| 159079 | Avh113 | + |  |  | 1.0000 | 20 | RFLR | EER |  |
| 159080 | Avh114 | + |  |  | 0.9990 | 23 | RHLK | DGEEAR |  |
| 159081 | Avh115 | + | + |  | 1.0000 | 20 | RHLR | DNDEER |  |
| 159088 | Avh125 | + |  |  | 0.9830 | 23 | RTLQ | ETEER |  |
| 159092 | Avh131 | + |  |  | 1.0000 | 22 | RRLR | DEER |  |
| 159094 | Avh137 | + | + |  | 1.0000 | 20 | RFLR | ESMTER |  |
| 159100 | Avh144 | + |  |  | 1.0000 | 21 | RLLR | DDEER |  |
| 159101 | Avh145 | + |  |  | 0.9990 | 23 | RLLR | DDEER |  |
| 159102 | Avh146 | + | + |  | 1.0000 | 20 | RLLR | EDEER |  |
| 159103 | Avh147 |  | + |  | 1.0000 | 19 | RFLR | DEDEER |  |
| 159104 | Avh148 | + |  |  | 1.0000 | 23 | RLLR | DAEER |  |
| 159105 | Avh149 | + | + |  | 0.9990 | 22 | RLLR | DDEER |  |
| 159109 | Avh153a1 | + |  |  | 1.0000 | 23 | RLLR | EDSEER |  |
| 159110 | Avh154 | + | + |  | 1.0000 | 24 | RLLR | DDEER |  |
| 159111 | Avh155 | + |  |  | 1.0000 | 26 | KLTTR | DENDEER |  |
| 159114 | Avh158 | + | + |  | 0.9990 | 29 | RHLR | DGVNSEEK |  |
| 159119 | Avh163 | + | + |  | 1.0000 | 23 | RMVR | DGDEDR |  |
| 159120 | Avh164 |  | + |  | 0.9960 | 28 | RHLR | EER |  |
| 159123 | Avh167 | + |  |  | 1.0000 | 17 | RNLR | DDEDR |  |
| 159124 | Avh168 | + |  |  | 1.0000 | 23 | RVLR | DKENEDR |  |
| 159127 | Avh171 |  | + |  | 0.9980 | 25 | RYLR | EEEER | Avr4/6^[15]^ |
| 159128 | Avh172 | + | + | + | 1.0000 | 20 | RLLR | DNEDR |  |
| 159129 | Avh173 |  | + |  | 0.9980 | 24 | RYLR | DEEEDEER |  |
| 159135 | Avh179 | + | + |  | 1.0000 | 20 | RKLE | DPAGEAR |  |
| 159136 | Avh180 |  | + |  | 1.0000 | 20 | RSLR | DEEER |  |
| 159137 | Avh181 | + | + |  | 1.0000 | 20 | KSSLR | DENDEER |  |
| 159138 | Avh182 | + |  |  | 1.0000 | 24 | RRLR | DEDEER |  |
| 159144 | Avh188 | + |  |  | 1.0000 | 21 | KNLR | DYGDGER |  |
| 159146 | Avh190 | + |  |  | 1.0000 | 21 | RNLR | NEER |  |
| 159150 | Avh194 | + |  | + | 0.9960 | 23 | RSLR | EDESSELR |  |
| 159152 | Avh196 | + | + | + | 1.0000 | 20 | RMLLR | DEEER |  |
| 159155 | Avh199 | + |  |  | 1.0000 | 20 | RLLR | DDEER |  |
| 159168 | Avh212 | + |  |  | 0.9990 | 21 | RMLV | KEER |  |
| 159171 | Avh215 |  |  | + | 0.9880 | 28 | RHLR | DRDEER |  |
| 159184 | Avh228 |  | + |  | 0.9990 | 24 | RKLK | EDEER |  |
| 159185 | Avh229 |  | + |  | 1.0000 | 25 | RFLR | EEER |  |
| 159188 | Avh232 |  | + |  | 0.9990 | 23 | RHLR | DEER |  |
| 159190 | Avh234 |  | + |  | 1.0000 | 20 | RLLR | DASDEDR |  |
| 159191 | Avh235 |  | + |  | 1.0000 | 22 | RLLR | DDDSEER |  |
| 159192 | Avh236 |  | + |  | 1.0000 | 22 | RLLR | DDDSEER |  |
| 159193 | Avh237 |  | + |  | 1.0000 | 22 | RLLR | DDDSEER |  |
| 159194 | Avh238 | + | + | + | 0.9990 | 19 | RFLR | DGKTEER |  |
| 159195 | PsRF239 |  | + |  | 0.9420 | 19 | RLLPR | DDQER |  |
| 159196 | Avh240 | + | + |  | 1.0000 | 23 | RHLR | DDEDER |  |
| 159197 | Avh241 | + |  |  | 1.0000 | 18 | RLLR | DTFEDR |  |
| 159198 | Avh242 |  | + |  | 1.0000 | 23 | RHLR | DEER |  |
| 159199 | Avh243 | + |  |  | 1.0000 | 20 | RFLR | EER |  |
| 159202 | Avh246 | + | + |  | 1.0000 | 24 | RHLK | DEEER |  |
| 159204 | Avh248 | + |  |  | 1.0000 | 20 | RSLR | DEER |  |
| 159205 | Avh249 | + |  |  | 1.0000 | 21 | RSLR | DADEER |  |
| 159208 | Avh252 | + |  |  | 1.0000 | 22 | RSLR | DEQR |  |
| 159212 | Avh256 | + | + |  | 0.9310 | 48 | RLLR | DLTEER |  |
| 159214 | Avh258 | + |  |  | 1.0000 | 24 | RHLK | DLTAESEER |  |
| 159216 | Avh260 |  | + | + | 0.9990 | 24 | RRLK | EEER |  |
| 159217 | Avh261 |  | + |  | 0.9990 | 24 | RHNLK | DSEER |  |
| 159219 | Avh263 |  | + |  | 1.0000 | 21 | RSLR | EAEER |  |
| 159225 | Avh269 | + |  |  | 1.0000 | 19 | RLLR | EER |  |
| 159226 | Avh270 | + | + | + | 1.0000 | 17 | RLLR | EEEEER |  |
| 159228 | Avh272 | + |  |  | 1.0000 | 20 | RLLR | DDEER |  |
| 159238 | Avh282 | + |  |  | 0.8340 | 24 | RSLR | EER |  |
| 159239 | Avh283 | + |  |  | 1.0000 | 21 | RLLQ | DSDER |  |
| 159240 | Avh284 | + |  |  | 1.0000 | 23 | RLLR | EDSEER |  |
| 159244 | Avh288 | + | + | + | 0.9990 | 20 | RRLK | DLTAHEEER |  |
| 159246 | Avh290 | + |  |  | 0.9940 | 24 | RSLR | DSSELR |  |
| 159248 | Avh292 | + |  |  | 1.0000 | 24 | RSLR | DDEDR |  |
| 159250 | Avh294 |  | + |  | 0.9920 | 21 | RHLK | DEER |  |
| 159263 | Avh307 | + |  |  | 1.0000 | 17 | RSLR | EER |  |
| 159286 | Avh330 | + |  |  | 0.8180 | 20 | RSLR | DANVSIENR |  |
| 159287 | Avh331 | + |  |  | 0.8180 | 20 | RSLR | DANVSIENR |  |
| 159291 | Avh335 |  | + |  | 0.9290 | 22 | RSLR | DKVEK |  |
| 159296 | Avh340 |  | + |  | 0.9970 | 20 | RQLR | DEER |  |
| 159302 | Avh346 | + |  |  | 0.9990 | 24 | RALR | EER |  |
| 159304 | Avh348 | + |  |  | 0.9990 | 21 | RVLK | DTKGDIER |  |
| 159325 | Avh370 | + |  |  | 0.9990 | 20 | RLLR | DEGDSPEDR |  |
| 159339 | Avh416 | + |  |  | 1.0000 | 23 | RVLR | DKENEDR |  |
| 159340 | Avh421 | + |  |  | 1.0000 | 20 | RTLR | DDNSEER |  |
| 159346 | Avh429 | + |  |  | 0.9940 | 20 | RLLS | DESEEER |  |
| 159357 | Avh441 | + |  |  | 0.9990 | 24 | RFLR |  |  |
| 159373 | Avh457 | + |  |  | 0.9950 | 44 | RFLR | DADSEDR |  |
| 159375 | Avh459 | + | + | + | 1.0000 | 21 | RFRR | DDEER |  |
| 159377 | Avh461 |  |  | + | 0.999 | 21 | RRLR | EIDR |  |
| 159378 | Avh462 |  | + |  | 0.9980 | 24 | RFLR | EEGEER |  |
| 159380 | Avh464 | + |  |  | 0.9990 | 24 | RLLR | DEAR |  |

**a:** Protein IDs of P6497 RXLR alleles; identical at www.jgi.doe.gov and vmd.vbi.vt.edu.

**b:** Suffixes a indicate paralogs encoding proteins with greater than 95% amino acid sequence identifty. Suffixes a1 indicate paralogs that encode identical proteins. PsRF genes indicate pseudogenes that in some cases were previously classified as functional genes.

**c:** Identification of expressed RXLR effector genes by examination of three kinds of gene expression data: DGE, digital gene expression data generated by Solexa sequencing [35]; Affy, soybean Affymetrix array data [12,31]; and ESTs [61], the expression sequence tags were obtained by BLAST in VBI *P. sojae* unigenes (VBI microbial database). “+” indicates that transcripts of the gene were detectable using the method. All these data were derived from *P. sojae* strain P6497 (*Rps*3b avirulent).

**d:** SP HMM (signal peptide prediction HMM probability) and SP length (signal peptide) were predicted using SignalP v3.0 (<http://www.cbs.dtu.dk/services/SignalP/>).

**e:** RXLR and dEER motifs were previously bioinformatically identified [42].
