# Supplementary material for: Phytophthora sojae Avirulence Effector Avr3b is a Secreted NADH and ADP-ribose Pyrophosphorylase that Modulates Plant Immunity
Source: PLoS Pathog. 2011 Nov 10;7(11):e1002353. doi: 10.1371/journal.ppat.1002353 (PMC3213090; doi:10.1371/journal.ppat.1002353)
Supplement: Table S2 — Cleaved Amplified Polymorphic (CAP) markers for Avr3b genotyping. (DOC) [file ppat.1002353.s005.doc]

**Table S2: Cleaved Amplified Polymorphic (CAP) markers for Avr3b genotyping**

| **Marker name** | **Primer sequences^a^** | **Restriction** | **Product size (bp)** | |
| --- | --- | --- | --- | --- |
|  |  | **Enzyme^b^** | **P6497** | **P7076** |
| CAPSAvh238 | 5'-GCCATGCGCGGTGTATTCTTCGTT-3' | EcoR V | 1139 | 360 + 779 |
|  | 5'-GCCTTCACCGGTCTTCTTCTTCTA-3' |  |  |  |
| CAPSAvh307 | 5'-CTGTTTCGACATCGCGAAGC-3 | Acl I | 1277 | 346 + 922 |
|  | 5‘-TCAAGTGAAATCTCTCTATTCC-3' |  |  |  |
| CAPSAvh113 | 5'-AGCAAGAAGCGCACGAAGAGC-3' | Sca I | 617 | 316 + 301 |
|  | 5'-TTGTCGGCGGAGGTGAGAAAG-3' |  |  |  |
| CAPSAvh258 | 5'-GCAGGTTTACGAAGAAGACGAGC-3' | Mlu I | 720 | 380 + 339 |
|  | 5'-AAGGAGACCAATGGTGCTGTTCG-3' |  |  |  |
| CAPSAvh20 | 5'-GTCACTCGCCTTGTGAGCTT-3' | - | 406 | 604 |
|  | 5'-GACGACGATACCAAAGCAGT-3' |  |  |  |
| CAPSAvh288 | 5'-TCAAGAGAGCCGACGACCTCAC-3' | Hind III | 799 | 339 + 460 |
|  | 5'-GAAACCATCGAGTTGAAGCCGG-3' |  |  |  |
| CAPSAvh6 | 5’-ATGTACTATGTACTGCGGGATC-3' | - | 555 | 0 |
|  | 5'-CTAATTCATGCGGTAGTGGATTT-3' |  |  |  |
| CAPSAvh307_ds6k | 5'-TTCTCACGAGCTAGATGCC-3' | Bfa I | 383 + 224 | 607 |
|  | 5'-CTAGCACGATTTTCTTCTCC-3' |  |  |  |
| CAPSAvh9_us10k | 5'-GATTACTTTCCCGAGACTACC-3' | Acl I | 758 | 409 + 349 |
|  | 5'-CCGTCTTCTTTTCAATGGAC-3' |  |  |  |

**a:** Primers were used to amplify PCR framents from *P. sojae* genomic DNA.

**b:** The restriction enzymes were used to digest the PCR products generated by the corresponding primers. ‘”-” represented that the marker does not require enzyme to digest.
