# Supplementary material for: Phytophthora sojae Avirulence Effector Avr3b is a Secreted NADH and ADP-ribose Pyrophosphorylase that Modulates Plant Immunity
Source: PLoS Pathog. 2011 Nov 10;7(11):e1002353. doi: 10.1371/journal.ppat.1002353 (PMC3213090; doi:10.1371/journal.ppat.1002353)
Supplement: Table S3 — RXLR effectors that are located in the Avh307 region. (DOC) [file ppat.1002353.s006.doc]

**Table S3: RXLR effectors that are located in the Avh307 region**

| **Name** | **Protein ID** | **Genomic position^a^** | **Expression (P6497)^b^** | **Polymorphism (P7076)^c^** |
| --- | --- | --- | --- | --- |
| *Avh*309 | 159265 | scaffold_3: 1257772-1257161 | Yes | No |
| *Avh*308 | 159264 | scaffold_3: 1296437-1295907 | No | Yes |
| *Avh*9 | 159000 | scaffold_3: 1434377-1434889 | No | No |
| *Avh*307 | 159263 | scaffold_3: 1520669-1521613 | Yes | Yes |
| *Avh*198 | 159154 | scaffold_3: 1735417-1735211 | No | No |
| *Avh*340 | 159296 | scaffold_3: 1900450-1900202 | Yes | No |
| *Avh*238 | 159194 | scaffold_3: 2234580-2234002 | Yes | Yes |
| *Avh*302 | 159258 | scaffold_3: 2316620-2316913 | No | No |

**a:** Genomic position information was from *P. sojae* genome assembly version 5.0 (VBI microbial database vmd.vbi.vt.edu).

**b:** RT-PCR was performed by using a cDNA library as a template; the cDNA library is a mixed cDNA library covering 10 different developmental and infection stages.

**c:** Amino acid polymorphism refers to the sequence polymorphism between avirulent strain (P6497) and virulent strain (P7076).
