# Supplementary material for: Phytophthora sojae Avirulence Effector Avr3b is a Secreted NADH and ADP-ribose Pyrophosphorylase that Modulates Plant Immunity
Source: PLoS Pathog. 2011 Nov 10;7(11):e1002353. doi: 10.1371/journal.ppat.1002353 (PMC3213090; doi:10.1371/journal.ppat.1002353)
Supplement: Table S4 — Predicted Phytophthora Nudix hydrolase RXLR effectors. (DOC) [file ppat.1002353.s007.doc]

**Table S4: Predicted *Phytophthora* Nudix hydrolase RXLR effectors**

| Avr3b^P6497^ | ***MRFLFLLVAVALASAEA***AESAAMIHEEAGVSSFNTLTGGNQIQ**RSLRVYLGANEER**GVGGVKLENILTIFVQRAKAKLPQGFTAAALGNWKGFSRRVDTVMEHYPKGLSEKAIKELRTAETKRFTDYAMLGPSDKYNLLRPMQGVDEAMIAPNLVSLTGRKNQVLGDAGGRSVVCNVVMRSEAEGGGILLISSSKLDKQDFILPK**GGLEKGEIAYGAAKREVLEEGGV**RYRSDIFIFYRWSVLTLVMALQVKVKKLKELGVTLVGDKTYESFLMRSKKVYEQWSESRRLRVWVRECQSIEIPGETQLTVRLLFN* |
| --- | --- |
| Avr3b^P7076^ | ***MRFLFLLVAVALASAEA***AESAAMIHEEAGVSSFKTLTGVNQIQ**RSLRVLLGADEER**GVGGVSLKNILAPFKQTFQHPIAITAAALRELNDFKSLVNTVMKHYPKGLSEKAVQELRTAEEKRLTDYVFLGASDKNKLLREMDGVDEAKIASNLKSLTGRENQVLGEAGGRSVVCNVVMRPEAEGGGILLISSSKLDKLDFILPK**GGLEKGEIAYKAAKREVLEEGGVR*** |
| PITG05846 | ***MRVLQLICLVALISRCAA***DTATTNSHTASTLKINSDADAVSRVLAADRRQAT**RSLRQFDHDELAGGDAEDQER**GISISGPVEKMVTKFRDGLNAVLSKNPVRLRASLVEQLSKTYSYADKLSTSTLKQLEHIEKLRAADIKKGVKGSKATPGGMRRNVEPFPGMSTVPRKFLESHVGRDGQRFGKDGSRLLSAGVVTRFNDKGEREMLMISSSNPKKREFLPPK**GGWDKGEDIKTAALREVIEEGGV**CGQLWSILVVDMLTYL* |
| PITG06308 | ***MQTIQLIIFVAFVLSRAAA***SISSFSDPTSIVNINHDANRLSRALAAGQNQTQ**RSLRQHEGEDR**GAIDKADEVVSKMKALMGTAKNVPNNLAALIAKRSKTAGEFVRRPFLVSKLSKRYNIADQLSFSTLKQLDKIDNMRIVDIKNGIKGNKKTPNGMRRKIKHFEGMKTAPQKFLESHVGRDMQRYGKDGSRWLSAGVVTRTTDQGERQILLISSSNPARGDFLLPK**GGWDRGEKIKKAALREVMEEGGV**CRAL* |
| PITG15679 | ***MKVLQLIALTALVSSCVAASAA***DPSGLAKTKSDVDVLSRVLADHEQTN**RSLRRYDLEGLDSVNSNREER**NSITMVDDVVTKASGLVDDVMGKTDDVVGKAGQFGKVPTKLRDVATKNMDKIKEMTARSALVKTLTGRYDYAEKLSLSALKQLDDIEKVRAVDIKKGIKGSKETPDGMRRVIEPFEGMKVAPKKFLESHVGRADQRYGKDGSRLLSANVVMRLNDKGEKQILLISSSNPKKGDFLLPK**GGWDKGEDVKKAALREVIEEGGV**R* |
| PITG15732 | ***MRVLLILFVLISSASA***TSSEHVGITNTERFPIAAEVH**RFLRRHYLEEEADIESDDEDR**GGLDKVDDLITKVDDALGITGKMDDVAGKLGKVHVAPTTKTAVEKMEHAGLVKHLSGKYSVADKLSLTTLRQLAKVDEQRLKDNRVFDKKTGSGMRKKIEPFEGMKIAPQKYLEAHVARAGQLVDKENNRLLSAVVIGDGDNVLLISSSKKPNDWILPK**GGWDHGEGIEKAALREVIEEAGV**RLFFCNVFLYIYKYFLLIVVLQIQARLNHDLGKFTYKDGDKGYGLFAYTMDDVQRFDDWAESSRYRIDVSRYAAKNSFCIIGKLTCV* |
| PrAvh165 | ***MRVLSLVALIAFVSSCEA***TSAVSNSDKVNVAKLDSGIASLPRALAEHNDQIK**RSLRRHDDEER**QYGTQLIDDVITKVDDVVTKATVAEQKLPSKWTSALKKFRLANPKTPMQIVRAKYPKGLSSSTMRQLVQTEIQRADDIANGVKMVKTGEYKMQRQIDEFPGMKNAPLLTSNTGRAEQLLADDGSRLLACIVASRSAEKGGGDVLLVSSSNARKNDWLLPK**GGWDKGETVEKAAMRELIEEGGV**R* |
| PrAvh268 | ***MRLLLVALIIAVSGCEA***ASVTTKSNVANSGSGISFVSRLDTHNDSKEAE**RSLHRDENASDEEER**GGGAEKLDEVMEKVVHASKKLRKRVSTAALEKLNDLAAKVKKVSPMRPENLSPSTLKQLDKIDEMKRLDRIAEAKRLDALPEAQRIAELAKKNPGKSTPDGMFRKMELGPDAKIAPTLTSEIGRAKQRFDDDGRRLLSCIVVSRRTEDGGGDVLLISSSNPKRDDWILPK**GGWNEGEGIEKAAWRELVEEGGV**RRLFC* |
| PrAvh281 | ***MRVLSLVALIALVSSCEA***ASAVSNSDKVEVAKLDSGIASLSRALAEHNDQIK**RSLRRHDYDELAEVDSGDGER**MGGAQLIDDVITKVDDVVTKATVAEQKLPSKWTSALKKFRLANPKTPMQIVRAKYPKGLSSSTMRQLVQTEIQRADDIANGVKMVKTGEYKMQRQIDEFPGMKNAPLLTSNTGRADQMLDGDNSRLLACIVASRSVEKGGGDVLLVSSSNARKNDWLLPK**GGWDKGETVEHAAMRELIEEGGV**R* |
| Pc102433 | ***MLLRAIVIAVAFVTISLVATGSEA***SSITVVDPVADRSTQVK**RSLRLRNLESVDEDR**GPIAGLEKVDDILTKTEKASRKAGKVPSGLKNLLLRNIDEFAEHSKLAKKLSGLKLYKDAGLEKMSLSTLRQLDDIEVKRVSDIKNGITGNKDTPGGMRRKMDHVVGDVAPAKYLTSHIGRGDQLYGADGSRLLSSAVVSRPAEQGGGKVLLISSSKPEKGDWLLPK**GGWDKGEDIETAALREVMEEGGV**RPVLFFLGLSYQYSN* |
| Bold and italics = signal peptide; Bold and underline = RXLR-dEER motif; Bold = Nudix hydrolase | |
