# Supplementary material for: Phytophthora sojae Avirulence Effector Avr3b is a Secreted NADH and ADP-ribose Pyrophosphorylase that Modulates Plant Immunity
Source: PLoS Pathog. 2011 Nov 10;7(11):e1002353. doi: 10.1371/journal.ppat.1002353 (PMC3213090; doi:10.1371/journal.ppat.1002353)
Supplement: Table S5 — The hydrolytic activities of both pure Avr3b protein and plant protein extract against nucleotide derivatives. (DOC) [file ppat.1002353.s008.doc]

**Table S5: The hydrolytic activities of both pure Avr3b protein and plant protein extract against nucleotide derivatives**

| **Substrates ^a^** | **Avr3b^P6497^ relative activity ^b^** | **Avr3b^QQQQ^ relative activity ^b^** |
| --- | --- | --- |
| **Immunoprecipitates ^c^** |  |  |
| NADH | 4.94 ± 1.16 ^e, f^ | 1.67 ± 0.62 ^e, f^ |
| ADPR | 4.32 ± 0.86 ^e, f^ | 1.51 ± 0.30 ^e, f^ |
| NADPH | 1.95 ± 0.55 ^e, f^ | 1.39 ± 0.07 ^e^ |
| NAD | 1.22 ± 0.19 | 1.09 ± 0.20 |
| Ap4A | 1.64 ± 0.07 ^e, f^ | 0.98 ± 0.12 |
| FAD | 1.94 ± 0.23 ^e, f^ | 0.99 ± 0.12 |
| Coenzyme A | 1.28 ± 0.06 ^e^ | 1.02 ± 0.11 |
| **Total extract** ^d^ |  |  |
| NADH | 2.07 ± 0.13 ^e, f^ | 1.24 ± 0.22 ^e, f^ |
| ADPR | 1.76 ± 0.24 ^e, f^ | 1.07 ± 0.12 |
| NADPH | 1.17 ± 0.08 | 1.18 ± 0.04 |
| NAD | 1.19 ± 0.05 ^e^ | 1.08 ± 0.04 |
| Ap4A | 1.07 ± 0.03 | 1.02 ± 0.07 |
| FAD | 1.00 ± 0.07 | 0.93 ± 0.04 |
| Coenzyme A | 1.03 ± 0.13 | 1.07 ± 0.27 |

**a:** The substrates tested in this paper included: nicotinamide adenine dinucleotide reduced form (NADH), adenosine diphosphate ribose (ADPR), Nicotinamide adenine dinucleotide phosphate reduced form (NADH), Nicotinamide adenine dinucleotide (NAD), Diadenosine tetraphosphate (Ap4A), flavin adenine dinucleotide (FAD), and Coenzyme A.

**b:** The hydrolase activity (A_820_ reading) of Avr3b samples (Avr3b^P6497^ or Avr3b^QQQQ^) relative to the activity of control samples (from GFP-expressing tissue).

**c:** Avr3b^P6497^ and Avr3b^QQQQ^ proteins eluted from immunoprecipitates as shown in Figure 4B.

**d:** Total protein extract from *N. benthamiana* tissue transiently expressing Avr3b^P6497^ or Avr3b^QQQQ^. Values are the averages of at least four replicates.

**e:** Significantly different than the GFP control, (student’s test, p < 0.05)

**f:** Significantly different than the GFP control, (Duncan’s multiple range testing p < 0.05)
