# Supplementary material for: Phytophthora sojae Avirulence Effector Avr3b is a Secreted NADH and ADP-ribose Pyrophosphorylase that Modulates Plant Immunity
Source: PLoS Pathog. 2011 Nov 10;7(11):e1002353. doi: 10.1371/journal.ppat.1002353 (PMC3213090; doi:10.1371/journal.ppat.1002353)
Supplement: Table S6 — List of P. sojae strains used in this study. (DOC) [file ppat.1002353.s009.doc]

**Table S6: List of *P. sojae* strains used in this study**

| **Race** | **Name** | **Virulence^a^**  ***Rps*3b** | **Origin^b^** | **From^c^** | **Avr3b sequence** |
| --- | --- | --- | --- | --- | --- |
| Race 1 | 48FPA18 | A | Ohio (F.S.) | F.S. | Avr3b^P6497^ |
| Race 2 | P6497 | A | Mississippi (B.K.) | B.M.T. | Avr3b^P6497^ |
| Race 3 | 25MEX4 | A | Ohio (F.S.) | F.S. | Avr3b^P6497^ |
| Race 7 | P7064 | A | Canada (C.M.) | B.M.T. | Avr3b^P6497^ |
| Race 8 | ACR8 | A | Unknown | T.A. | Avr3b^P6497^ |
| Race 9 | ACR9 | A | Harrow, ON (C.M.) | T.A. | Avr3b^P6497^ |
| Race 10 | ACR10 | A | Stoneville, MS (B.K.) | T.A. | Avr3b^P6497^ |
| Race 11 | ACR11 | A | Harrow, ON (C.M.) | T.A. | Avr3b^P6497^ |
| Race 12 | ACR12 | V | Stoneville, MS (B.K.) | T.A. | Avr3b^P7076^ |
| Race 14 | R14 | A | Unknown | B.M.T. | Avr3b^P6497^ |
| Race 16 | ACR16 | A | Stoneville, MS (B.K.) | T.A. | Avr3b^P6497^ |
| Race 17 | P7074 | V | Stoneville, MS (B.K.) | T.A. | Avr3b^P7076^ |
| Race 19 | P7076 | V | Stoneville, MS (B.K.) | B.M.T. | Avr3b^P7076^ |
| Race 20 | ACR20 | V | Unknown | T.A. | Avr3b^P7076^ |
| Race 21 | ACR21 | A | Lafayette, IN (F.A.L) | T.A. | Avr3b^P6497^ |
| Race 24 | ACR24 | A | Lafayette, IN (F.A.L) | T.A. | Avr3b^P6497^ |
| Race 25 | ACR25 | A | Lafayette, IN (F.A.L) | T.A. | Avr3b^P6497^ |
| Race 31 | R31 | A | Unknown | B.M.T. | Avr3b^P6497^ |
| - | HN25 | A | China (L.K.C.) | NJAU collection | Avr3b^P6497^ |
| - | HN35 | A | China (L.K.C.) | NJAU collection | Avr3b^P6497^ |

**a:** Virulence on soybean Rps3b; A, avirulent; V, virulent.

**b:** Original site of isolation and, when known, investigator who conducted the isolation.

**c:** Investigator who provided the culture. T.A. = T. Anderson (Agriculture and Agri-Food Canada, Harrow, ON); B.K.= B. Keeling (USDA, Stoneville, MS); F.A.L.= F.A. Laviolette (Purdue University, West Lafayette, IN); C.M = C. Meharg (Agriculture and Agri-Food Canada, Harrow, ON); F.S.= F. Schmitthenner (Ohio State University, Wooster, OH); B.M.T. = B.M. Tyler (Virginia Bioinformatics Institute, Blacksburg, VA); L.K.C. = Linkai Cui (Nanjing Agricultural University, China).
