# Supplementary material for: Phytophthora sojae Avirulence Effector Avr3b is a Secreted NADH and ADP-ribose Pyrophosphorylase that Modulates Plant Immunity
Source: PLoS Pathog. 2011 Nov 10;7(11):e1002353. doi: 10.1371/journal.ppat.1002353 (PMC3213090; doi:10.1371/journal.ppat.1002353)
Supplement: Table S7 — Sequences referred in this paper. (DOC) [file ppat.1002353.s010.doc]

**Table S7: Sequences referred in this paper.**

| **Gene Name** | **NCBI Accession numbers ^a^** | **Origin** |
| --- | --- | --- |
| Avr3b^P6497^ | JF892553 | *P. sojae* |
| Avr3b^P7076^ | JF892554 | *P. sojae* |
| PrAvh165 | JF892555 | *P. ramorum* |
| PrAvh268 | JF892556 | *P. ramorum* |
| PrAvh281 | JF892557 | *P. ramorum* |
| PITG05846 | JF892558 | *P. infestans* |
| PITG06308 | JF892559 | *P. infestans* |
| PITG15679 | JF892560 | *P. infestans* |
| PITG15732 | JF892561 | *P. infestans* |
| Pc102433 | JF892562 | *P. capsici* |
| AtNUDT7 | Q9SU14 | *A. thaliana* |

**a**: NCBI refers to National Center for Biotechnology Information at [www.ncbi.nih.gov](http://www.ncbi.nih.gov/).
